# Upper Respiratory Tract Co-detection of Human Endemic Coronaviruses and High-density Pneumococcus Associated With Increased Severity Among HIV-Uninfected Children Under 5 Years Old in the PERCH Study

**DOI:** 10.1097/INF.0000000000003139

**Published:** 2021-04-19

**Authors:** Daniel E. Park, Melissa M. Higdon, Christine Prosperi, Henry C. Baggett, W. Abdullah Brooks, Daniel R. Feikin, Laura L. Hammitt, Steve R. C. Howie, Karen L. Kotloff, Orin S. Levine, Shabir A. Madhi, David R. Murdoch, Katherine L. O’Brien, J. Anthony G. Scott, Donald M. Thea, Martin Antonio, Juliet O. Awori, Vicky L. Baillie, Charatdao Bunthi, Geoffrey Kwenda, Grant A. Mackenzie, David P. Moore, Susan C. Morpeth, Lawrence Mwananyanda, Wantana Paveenkittiporn, Mohammed Ziaur Rahman, Mustafizur Rahman, Julia Rhodes, Samba O. Sow, Milagritos D. Tapia, Maria Deloria Knoll

**Affiliations:** From the * Department of International Health, International Vaccine Access Center, Johns Hopkins Bloomberg School of Public Health, Baltimore, Maryland; † Department of Environmental and Occupational Health, Milken Institute School of Public Health, George Washington University, Washington, District of Columbia; ‡ Division of Global Health Protection, Centers for Disease Control and Prevention, Atlanta, Georgia; § Department of International Health, Johns Hopkins Bloomberg School of Public Health, Baltimore, Maryland; ¶ International Centre for Diarrhoeal Disease Research, Bangladesh (icddr,b), Bangladesh; ∥ Medical Research Council Unit, Basse, The Gambia; ** Department of Paediatrics, University of Auckland, New Zealand; †† Department of Pediatrics and Department of Medicine, Center for Vaccine Development and Global Health, University of Maryland School of Medicine, Baltimore, Maryland; ‡‡ Bill & Melinda Gates Foundation, Seattle, Washington; §§ Medical Research Council: Respiratory and Meningeal Pathogens Research Unit; ¶¶ Department of Science and Technology/National Research Foundation: Vaccine Preventable Diseases Unit, University of the Witwatersrand, Johannesburg, South Africa; ∥∥ Department of Pathology and Biomedical Sciences, University of Otago; *** Microbiology Unit, Canterbury Health Laboratories, Christchurch, New Zealand; ††† KEMRI Wellcome Trust Research Programme, Centre for Geographic Medicine Research, Coast, Kilifi, Kenya; ‡‡‡ Department of Infectious Disease Epidemiology, London School of Hygiene and Tropical Medicine, London, United Kingdom; §§§ Department of Global Health and Development, Boston University School of Public Health, Boston, Massachusetts; ¶¶¶ Department of Pathogen Molecular Biology, London School of Hygiene and Tropical Medicine; ∥∥∥ Microbiology and Infection Unit, Warwick Medical School, University of Warwick, Coventry, United Kingdom; **** Division of Global Health Protection, Thailand Ministry of Public Health–US Centers for Disease Control and Prevention Collaboration, Nonthaburi, Thailand; †††† Right to Care-Zambia; ‡‡‡‡ Department of Biomedical Sciences, School of Health Sciences, University of Zambia, Lusaka, Zambia; §§§§ Murdoch Children’s Research Institute, Melbourne, Australia; ¶¶¶¶ London School of Hygiene and Tropical Medicine, London, United Kingdom; ∥∥∥∥ Department of Paediatrics, University of Melbourne, Australia; ***** Department of Paediatrics & Child Health, Chris Hani Baragwanath Academic Hospital and University of the Witwatersrand, South Africa; ††††† Microbiology Laboratory, Middlemore Hospital, Counties Manukau District Health Board, Auckland, New Zealand; ‡‡‡‡‡ EQUIP-Zambia, Lusaka, Zambia; §§§§§ National Institute of Health, Ministry of Public Health, Nonthaburi, Thailand; ¶¶¶¶¶ Virology Laboratory, International Centre for Diarrhoeal Disease Research, Bangladesh (icddr,b), Bangladesh; ∥∥∥∥∥ Centre pour le Développement des Vaccins (CVD-Mali), Bamako, Mali; ****** Department of Pediatrics, Center for Vaccine Development and Global Health, University of Maryland School of Medicine, Baltimore, Maryland.

**Keywords:** coronavirus, pneumococcus, coinfection, pneumonia, COVID-19

## Abstract

Severity of viral respiratory illnesses can be increased with bacterial coinfection and can vary by sex, but influence of coinfection and sex on human endemic coronavirus (CoV) species, which generally cause mild to moderate respiratory illness, is unknown. We evaluated CoV and pneumococcal co-detection by sex in childhood pneumonia.

**Methods:**

In the 2011–2014 Pneumonia Etiology Research for Child Health study, nasopharyngeal and oropharyngeal (NP/OP) swabs and other samples were collected from 3981 children <5 years hospitalized with severe or very severe pneumonia in 7 countries. Severity by NP/OP detection status of CoV (NL63, 229E, OC43 or HKU1) and high-density (≥6.9 log_10_ copies/mL) pneumococcus (HDSpn) by real-time polymerase chain reaction was assessed by sex using logistic regression adjusted for age and site.

**Results:**

There were 43 (1.1%) CoV+/HDSpn+, 247 CoV+/HDSpn−, 449 CoV−/HDSpn+ and 3149 CoV−/HDSpn− cases with no significant difference in co-detection frequency by sex (range 51.2%–64.0% male, *P* = 0.06). More CoV+/HDSpn+ pneumonia was very severe compared with other groups for both males (13/22, 59.1% versus range 29.1%–34.7%, *P* = 0.04) and females (10/21, 47.6% versus 32.5%–43.5%, *P* = 0.009), but only male CoV+/HDSpn+ required supplemental oxygen more frequently (45.0% versus 20.6%–28.6%, *P* < 0.001) and had higher mortality (35.0% versus 5.3%–7.1%, *P* = 0.004) than other groups. For females with CoV+/HDSpn+, supplemental oxygen was 25.0% versus 24.8%–33.3% (*P* = 0.58) and mortality was 10.0% versus 9.2%–12.9% (*P* = 0.69).

**Conclusions:**

Co-detection of endemic CoV and HDSpn was rare in children hospitalized with pneumonia, but associated with higher severity and mortality in males. Findings may warrant investigation of differences in severity by sex with co-detection of HDSpn and SARS-CoV-2.

## Abstract 2

Supplemental Digital Content is available in the text.

Bacterial coinfection increased morbidity and mortality in both the 1918 and 2009 influenza A pandemics.^1,2^ By some estimates, >95% of the deaths during the 1918 influenza pandemic involved complication with bacterial pneumonia, most commonly with *Streptococcus pneumoniae*.^1,3^ In a large-scale clinical study across the United States during the 2009 H1N1 pandemic, 28% of H1N1 2009 virus-positive samples had at least 1 other pathogen detected.^4^ In nonpandemic contexts, pneumonia etiology studies have attributed around 10%–30% of hospitalized pneumonia to multiple pathogens, particularly to coinfection with respiratory viruses and pneumococcus.^5–8^

In late 2019, a novel enveloped RNA coronavirus (CoV) designated SARS-CoV-2 emerged and proliferated globally, causing the associated illness named the 2019 coronavirus disease (COVID-19).^9–12^ While most COVID-19 cases were adults, severe disease and mortality have been reported in children.^13,14^ Initial studies suggest that pneumococcal coinfection with COVID-19 is relatively rare^15,16^; however, diagnosing coinfection remains challenging because similar clinical presentation and poor sensitivity of detecting pneumococcal pneumonia. In adults, pneumococcal pneumonia diagnosis relies on sputum and bronchoscopy, which have been restricted during the pandemic,^16–18^ and blood cultures which only detect approximately 25% of cases.^19^ Prior studies have suggested that high-density detection of pneumococcus in the nasopharynx or oropharynx may be an informative proxy for pneumococcal pneumonia and help differentiate disease from colonization.^20,21^ Pneumococcal carriage itself may also contribute to severity, as high-density carriage of *S. pneumoniae* has been associated with immunologic priming, upper respiratory tract microbiome dysbiosis and increased susceptibility to viral coinfection.^22,23^

Differences in severity of disease by sex have been observed for influenza, SARS-CoV-2 and pneumococcus. Severity of influenza disease and COVID-19 is generally greater in males, including in male children.^24,25^ Incidence and severity of invasive pneumococcal disease (IPD) is also higher in males.^26–29^ The relative contribution of behavioral and biologic causes for sex differences is unknown.^25,30–32^

Other endemic CoV species have received less attention than SARS-CoV-2 because most infections are asymptomatic or mild, although severe disease and mortality have been reported in both children and adults.^13,14^ Endemic CoVs commonly found in human circulation include CoV-NL63, CoV-229E, CoV-HKU1 and CoV-OC43, of which CoV-HKU1 and CoV-OC43 are more closely related to SARS-CoV-2.^12^ There is in vitro evidence for co-pathogenesis of endemic CoV with pneumococcus,^33^ and in vivo data from a pneumococcal conjugate vaccine trial suggesting that pneumococcus may play a role in severe endemic CoV infections.^34^ However, few studies have examined evidence for co-pathogenesis at the population level.^7^ Endemic CoVs have been commonly found in children, both in healthy children and those hospitalized with pneumonia, but are not estimated to be a common cause of pneumonia.^35^

The Pneumonia Etiology Research for Child Health (PERCH) study evaluated the causes of hospitalized severe or very severe pneumonia in children in 7 developing countries, and included community controls to evaluate the background prevalence of infection in children without pneumonia.^35^ To explore severity of pneumonia associated with *S. pneumoniae* and endemic CoV coinfection and evaluate differences by sex, we evaluated the clinical and epidemiologic characteristics of NP/OP co-detection in the PERCH study.

## MATERIALS AND METHODS

PERCH enrollment occurred between August 2011 and November 2014 for 24 months at each of 9 study sites in 7 countries: Dhaka and Matlab, Bangladesh; Basse, The Gambia; Kilifi, Kenya; Bamako, Mali; Soweto, South Africa; Nakhon Phanom and Sa Kaeo, Thailand; and Lusaka, Zambia. Identification and selection of cases and controls have been described previously.^36^ Cases were children 28 days–59 months of age hospitalized with severe or very severe pneumonia (pre-2013 WHO definition). Severe pneumonia was defined as having cough or difficulty breathing and lower chest wall indrawing; very severe pneumonia was defined as cough or difficulty breathing and at least one of the following danger signs: central cyanosis, difficulty breast-feeding/drinking, vomiting everything, convulsions, lethargy, unconsciousness or head nodding. Exclusion criteria for cases were hospitalization within the previous 14 days, having been discharged as a PERCH case within the past 30 days, not residing in the study catchment area, or resolution of lower chest wall indrawing following bronchodilator therapy for those with wheeze.^37^ Controls were children randomly selected from the same communities as cases without symptoms of severe or very severe pneumonia who were frequency-matched by age-group and month of enrollment to the cases. Known HIV-positive participants were excluded from this analysis; children with unknown HIV-status from sites with low HIV prevalence were included. The study protocol was approved by the Institutional Review Boards or Ethical Review Committees at all 7 institutions and at The Johns Hopkins School of Public Health.

Sample collection methods have been described previously.^38,39^ In brief, a flocked nasopharyngeal (NP) swab (flexible minitip, Copan) and a rayon oropharyngeal (OP) swab specimen were collected from each case and control at enrollment and placed pooled into the same 3 mL vial of universal transport media (Copan). The NP/OP specimen was tested for pneumococcus (lytA gene target) and coronaviruses NL63, 229E, OC43 and HKU1 as part of a multiplex real-time polymerase chain reaction (PCR) assay (FTD Respiratory Pathogens 33, Fast-track Diagnostics, Sliema, Malta). Colonization density was quantified in copies per mL by applying standard curves from standards of known quantities. Pathogen-specific high-density thresholds were determined for common bacterial colonizers, including pneumococcus. The threshold of upper respiratory tract carriage density that best distinguished known pneumococcal cases from controls was ≥6.9 log copies/mL.^20,40,41^

Clinical characterization of the illness in cases was assessed at admission. Digital chest radiograph images were assessed by members of a panel of 14 radiologists and pediatricians who were trained in the standardized interpretation of pediatric chest radiographs.^42^

Coinfection status for primary analyses were defined using NP/OP detection as follows: coronavirus with high-density *S. pneumoniae* (CoV+/HDSpn+), coronavirus without high-density *S. pneumoniae* (CoV+/HDSpn−), HDSpn without coronavirus (CoV−/HDSpn+) and neither HDSpn nor coronavirus (CoV−/HDSpn−). A secondary analysis evaluated NP/OP CoV co-detection with 3 categories of *S. pneumoniae* density: (1) no *S. pneumoniae*; (2) low-density pneumococcus (<6.9 log_10_ copies/mL); and (3) high-density pneumococcus. Prevalence of co-detection in cases was compared with controls. We evaluated associations between density of CoV and pneumococcal density category, sex and mortality. To assess whether findings were unique to CoV and *S. pneumoniae* co-detections, supplemental analyses evaluated co-detection of CoV with *Haemophilus influenzae* and *Staphylococcus aureus*, and co-detection of *S. pneumoniae* with influenza A, B or C, human metapneumovirus, parainfluenzavirus 1 or 3 (Para 1/3) and respiratory syncytial virus A and B (RSV A/B). A sensitivity analysis was conducted to expand the definition of CoV+ to include CoV detected in induced sputum by PCR and the definition of HDSpn+ to include IPD cases that fell below the threshold, that is, had *S. pneumoniae* recovered from blood by culture or from lung aspirate or pleural fluid by culture or PCR. A second sensitivity analysis lowered the pneumococcal density threshold to 6.6 log_10_ copies/mL, which better aligned with detection of pneumococcal pneumonia from children with prior antibiotic use.^20^

### Statistical Analysis

Demographic, clinical and laboratory characteristics were compared by co-detection category using logistic regression adjusted for age and site for categorical variables or the Wilcoxon signed-rank test for continuous variables, with and without stratifying by sex. Wilson score intervals were used to generate binomial proportional confidence intervals (CIs). Certain models were only adjusted for age and subregion (Asia, Western Africa, Southern Africa and Eastern Africa) due to sample size limitations. Interaction terms for sex were included in regression models to test for differences in association between co-detection and covariates by sex. Other pathogen combinations were selected based on prior evidence in the literature and through Random Forest models to evaluate all potential pathogens as predictors of CoV detection. Statistical analyses were conducted in SAS, version 9.4, and R, version 3.3.1.

## RESULTS

Of 3888 HIV-negative cases enrolled between 2011 and 2014 with WHO-defined severe or very severe pneumonia with available NP/OP results, 7.5% (n = 290) had endemic coronavirus detected by NP/OP PCR (2.2% NL63, 1.1% 229E, 2.8% OC43 and 1.6% HKU1) and 492 (12.6%) had HDSpn detected. CoV+/HDSpn+ was observed in 43 (1.1%) cases, CoV+/HDSpn− in 247 (6.4%), CoV−/HDSpn+ in 449 (11.5%) and 3149 (81.0%) had neither. *S. pneumoniae* was detected by blood culture more frequently in HDSpn+ cases and most frequently in those also CoV+: CoV+/HDSpn+ 7.1%, CoV−/HDSpn+ 4.3%, CoV+/HDSpn− 1.2% and CoV−/HDSpn− 0.3% (Table S12, Supplemental Digital Content 1, http://links.lww.com/INF/E363). Controls (n = 4977) had higher CoV prevalence than cases (10.0% versus 7.5%), but CoV+/HDSpn+ prevalence was similar between cases and controls (1.1% versus 0.9%) (Table 1).

**TABLE 1.** Distribution of Human Endemic Coronavirus (CoV-NL63, CoV-229E, CoV-OC43 or CoV-HKU1) and HDSpn Co-detection Status in NP/OP by PERCH Case-control Status

Characteristic	Case (n = 3888)	Control (n = 4977)	aOR*; Case vs. Control (Ref)	*P*
Coronavirus positive, n	290	501		
Prevalence, % (95% CI)	7.5 (6.7–8.3)	10.1 (9.3–10.9)	0.67 (0.58–0.78)	<0.001
High-density Spn, n	492	379		
Prevalence, % (95% CI)	12.7 (11.7–13.7)	7.6 (6.9–8.4)	1.62 (1.40–1.87)	<0.001
Co-detection category				
CoV+/HDSpn+, n	43	47		
Prevalence, % (95% CI)	1.1 (0.8–1.5)	0.9 (0.7–1.3)	1.09 (0.71–1.66)	0.698
CoV+/HDSpn−, n	247	454		
Prevalence, % (95% CI)	6.4 (5.6–7.2)	9.1 (8.4–10.0)	0.63 (0.54–0.75)	<0.001
CoV-/HDSpn+, n	449	332		
Prevalence, % (95% CI)	11.5 (10.6–12.6)	6.7 (6.0–7.4)	1.69 (1.45–1.97)	<0.001
CoV-/HDSpn−, n	3149	4143		
Prevalence, % (95% CI)	81.0 (79.7–82.2)	83.3 (82.3–84.3)	0.96 (0.86–1.07)	0.433

* Odds ratio for case status compared with control, adjusted for age in months and site.

CoV indicates coronavirus; HDSpn, high-density *Streptococcus pneumoniae*; PERCH, Pneumonia Etiology Research for Child Health study; NP/OP, nasopharyngeal/oropharyngeal.

### Co-detection of Endemic CoV and HDSpn

Co-detection was not associated with age, sex or pneumococcal conjugate vaccination status, but CoV+/HDSpn+ cases were disproportionately from Mali (Table 2) (Supplemental Digital Content 1 and 2, http://links.lww.com/INF/E363). Cases with high pneumococcal density, with or without CoV, were half as likely to have received antibiotics before NP/OP swab collection compared with cases without high-density pneumococcus (24.4% versus 47.4%, *P* < 0.001).

**TABLE 2.** Characteristics of Children Hospitalized with Severe or Very Severe Pneumonia by NP/OP Co-detection Status of Endemic Coronavirus (CoV-NL63, CoV-229E, CoV-OC43 or CoV-HKU1) and HDSpn*

Characteristic	No. (% with Available Information)				*P*†
	A. CoV+/HDSpn+	B. CoV+/HDSpn−	C. CoV−/HDSpn+	D. CoV− and HDSpn−	
Total	43 (100)	247 (100)	449 (100)	3149 (100)	
Age <1 yr	27 (62.8)	174 (70.4)	284 (63.3)	1980 (62.9)	0.129
Male sex	22 (51.2)	158 (64.0)	242 (53.9)	1826 (58.0)	0.058
Site					<0.001
Bangladesh	5 (11.6)	33 (13.4)	63 (14.0)	424 (13.5)	
Thailand	0 (0)	10 (4.0)	3 (0.7)	208 (6.6)	
Mali	18 (41.9)	53 (21.5)	148 (33.0)	431 (13.7)	
The Gambia	9 (20.9)	48 (19.4)	95 (21.2)	457 (14.5)	
South Africa	6 (14.0)	54 (21.9)	81 (18.0)	654 (20.8)	
Zambia	3 (7.0)	25 (10.1)	31 (6.9)	401 (12.7)	
Kenya	2 (4.7)	24 (9.7)	28 (6.2)	574 (18.2)	
PCV vaccination status‡					
No. doses					0.226
0	14 (35.0)	91 (38.2)	165 (37.8)	1359 (44.5)	
1–2	10 (25.0)	80 (33.6)	128 (29.4)	787 (25.8)	
≥3	16 (40.0)	67 (28.2)	143 (32.8)	910 (29.8)	
Fully vaccinated for age	22 (55.0)	129 (54.2)	235 (53.9)	1456 (47.6)	0.307
Antibiotics received before NP/OP collection§	7 (16.3)	113 (45.7)	113 (25.2)	1498 (47.6)	<0.001

* ≥6.9 log copies/mL.

† *P* values for age in months obtained from the Wilcoxon test. The overall *P* value obtained from multinomial logistic regression adjusted for age and site (where applicable).

‡ During the study, PCV was in routine use in Kenya (introduced February, 2011), The Gambia (August, 2009), Mali (March, 2011) and South Africa (April, 2009); PCV was introduced in Zambia in July, 2013 (Lusaka), 3 months before the end of study enrollment. For children younger than 1 year, full vaccination was defined as having received at least 1 dose and being up to date for age on the basis of the child’s age at enrollment, doses received and country schedule (allowing a 4-week window for each dose); for children 1 year or older in all sites except Kenya, full vaccination was defined as having received three or more doses; for children older than 1 year in Kenya (which introduced PCV with catch-up campaign), full vaccination was defined as having received three or more doses, two doses if given at least 8 weeks apart and the child was older than 1 year of age at first dose, and one dose if the child was older than 2 years at any dose or at introduction.

§ Defined as serum bioassay positive, antibiotics administered at the referral facility or antibiotic administration before the collection of NP/OP PCR specimens at the study facility.

CoV indicates coronavirus; HDSpn, high-density *Streptococcus pneumoniae*; PERCH, Pneumonia Etiology Research for Child Health; PCV, pneumococcal conjugate vaccine; NP/OP, nasopharyngeal/oropharyngeal.

The association between the case co-detection group and clinical signs and symptoms at admission differed by gender (Table 3). For males only, CoV+/HDSpn+ cases were significantly more likely than the other co-detection groups to have WHO-defined very severe pneumonia (59.1% versus range 29.1%–34.7%, *P* = 0.04), require supplemental oxygen (45.0% versus 20.6%–28.6%, *P* < 0.001), have an mid-upper arm circumference-for-age Z-score < −3 SD (21.4% versus 2.6%–14.6%, *P* = 0.005). Among females only, leukocytosis was highest in CoV+/HDSpn+ cases (*P* = 0.01; interaction by sex *P* = 0.06) and an abnormal chest radiograph was least common among CoV+/HDSpn+ cases (28.6%) compared with the other co-detection groups (53.4%–65.8%, *P* = 0.03); for males, the abnormal chest radiograph was more common in HDSpn+ cases regardless of CoV status (64.7% and 58.1% for CoV+ and CoV−, respectively) compared with HDSpn− cases (43.8% and 49.4%, respectively; *P* = 0.03; interaction by sex *P* = 0.04). Other sex differences included less tachypnea among CoV+ in males (*P* = 0.009) but no difference among co-detection groups in females (interaction by sex *P* = 0.04). Fever at admission was common in all groups, but in males was more common among HDSpn+ cases (92.7%) compared with HDSpn− cases (79.8%, *P* = 0.03), whereas fever in females was most common in CoV−/HDSpn+ cases (90.8%) compared with other groups (range 80.9%–81.0%, *P* = 0.02; interaction by sex *P* = 0.99). Median C-reactive protein was highest among CoV+/HDSpn+ cases in males (44.3 versus 14.6–22.6 mg/L, *P* < 0.001) and was highest in HDSpn+ cases regardless of CoV status for females (Supplemental Digital Content 3 and 4, http://links.lww.com/INF/E363).

**TABLE 3.** Clinical Characteristics at Admission of Children Hospitalized with Severe or Very Severe Pneumonia by NP/OP Co-detection Status of Endemic Coronavirus (CoV-NL63, CoV-229E, CoV-OC43 or CoV-HKU1) and HDSpn*, by Sex

Characteristics	No. (% with Available Information)					
	A.	B.	C.	D.		*P*† for Interaction by Sex
	CoV+/HDSpn+, n = 43	CoV+/HDSpn−, n = 247	CoV−/HDSpn+, n = 449	CoV−/HDSpn−, n = 3149	Adjusted P*	
Male	22 (51.2)	158 (64.0)	242 (53.9)	1826 (58.0)		
Female	21 (48.8)	89 (36.0)	207 (46.1)	1323 (42.0)		
Very severe pneumonia (2005 WHO definition)	23	81	174	969	**<0.001**	0.335
	(53.5)	(32.8)	(38.8)	(30.8)		
Male	13	46	84	539	**0.039**	
	(59.1)	(29.1)	(34.7)	(29.5)		
Female	10	35	90	430	**0.009**	
	(47.6)	(39.3)	(43.5)	(32.5)		
Hypoxemia at admission	17	90	183	1098	0.061	0.873
	(39.5)	(36.6)	(40.9)	(35.0)		
Male	8	49	91	592	0.182	
	(36.4)	(31.0)	(37.6)	(32.5)		
Female	9	41	92	506	0.265	
	(42.9)	(46.6)	(44.7)	(38.3)		
WHO defined very severe or hypoxemia	30	130	254	1601	**0.002**	0.697
	(69.8)	(52.9)	(56.6)	(50.9)		
Male	16	74	125	885	0.094	
	(72.7)	(46.8)	(51.7)	(48.5)		
Female	14	56	129	716	**0.015**	
	(66.7)	(63.6)	(62.3)	(54.2)		
Supplemental oxygen (ever)‡	13/36	48/192	99/367	687/2475	**0.001**	0.240
	(36.11)	(25.0)	(27.0)	(27.8)		
Male	9/20	26/126	59/206	373/1460	**<0.001**	
	(45.0)	(20.6)	(28.6)	(25.5)		
Female	4/16	22/66	40/161	314/1015	0.584	
	(25.0)	(33.3)	(24.8)	(30.9)		
Tachypnea	38	199	398	2546	**0.044**	**0.036**
	(88.4)	(81.2)	(88.6)	(81.5)		
Male	17	122	220	1479	**0.009**	
	(77.3)	(77.2)	(90.9)	(81.5)		
Female	21	77	178	1067	0.423	
	(100.0)	(88.5)	(86.0)	(81.5)		
Fever	37	193	400	2533	**0.001**	0.986
	(86.1)	(78.1)	(89.1)	(80.4)		
Male	20	121	212	1462	**0.025**	
	(90.9)	(76.6)	(87.6)	(80.1)		
Female	17	72	188	1071	**0.017**	
	(81.0)	(80.9)	(90.8)	(81.0)		
Observed cough	22	168	302	2215	0.175	0.597
	(51.2)	(68.0)	(67.4)	(70.6)		
Male	10	110	167	1310	0.204	
	(45.5)	(69.6)	(69.0)	(71.9)		
Female	12	58	135	905	0.720	
	(57.1)	(65.2)	(65.5)	(68.7)		
Vomiting	12	49	104	643	0.382	0.109
	(28.6)	(19.8)	(23.2)	(20.4)		
Male	9	28	52	358	0.133	
	(40.9)	(17.7)	(21.5)	(19.6)		
Female	3	21	52	285	0.726	
	(15.0)	(23.6)	(25.1)	(21.5)		
Diarrhea	9	38	86	439	0.062	0.380
	(20.9)	(15.4)	(19.2)	(13.9)		
Male	5	27	53	248	**0.004**	
	(22.7)	(17.1)	(22.0)	(13.6)		
Female	4	11	33	191	0.816	
	(19.1)	(12.4)	(16.0)	(14.4)		
Abnormal chest radiograph‡	15/31	110/213	220/377	1381/2706	0.077	**0.043**
	(48.4)	(51.6)	(58.4)	(51.0)		
Male	11/17	60/137	119/205	783/1585	**0.030**	
	(64.7)	(43.8)	(58.1)	49.4)		
Female	4/14	50/76	101/172	598/1121	**0.030**	
	(28.6)	(65.8)	(58.7)	(53.4)		
Weight-for-height Z-score < −3 SDs	5	25	73	338	0.118	0.077
	(11.9)	(10.6)	(16.6)	(11.1)		
Male	4	19	43	184	0.052	
	(19.1)	(12.3)	(18.1)	(10.4)		
Female	1	6	30	154	0.247	
	(4.8)	(7.3)	(14.9)	(12.1)		
Weight-for-age Z-score < −3 SDs	7	31	84	491	0.216	**0.030**
	(16.7)	(12.7)	(18.7)	(15.7)		
Male	6	18	54	280	**0.014**	
	(28.6)	(11.5)	(22.3)	(15.4)		
Female	1	13	30	212	0.604	
	(4.8)	(14.8)	(14.5)	(16.1)		
Mid-upper arm circumference for age Z-score < −3 SDs§	4	6	34	120	0.172	0.339
	(15.4)	(4.6)	(12.8)	(6.5)		
Male	3	2	21	67	**0.005**	
	(21.4)	(2.6)	(14.6)	(6.4)		
Female	1	4	13	53	0.828	
	(8.3)	(7.4)	(10.7)	(6.8)		
Severe acute malnutrition¶	7	43	97	556	0.635	**0.024**
	(16.3)	(17.7)	(21.8)	(17.8)		
Male	6	26	55	295	0.298	
	(27.3)	(16.6)	(22.9)	(16.3)		
Female	1	17	42	261	0.308	
	(4.8)	(19.8)	(20.5)	(20.0)		
Height-for-age Z-score < −3 SDs	8	29	67	522	0.192	0.196
	(21.1)	(14.2)	(18.1)	(19.8)		
Male	6	21	37	329	0.234	
	(30.0)	(15.7)	(18.9)	(21.5)		
Female	2	8	30	193	0.427	
	(11.1)	(11.4)	(17.1)	(17.4)		
Leukocytosis	22	114	176	1273	**0.004**	0.057
	(55.0)	(49.1)	(42.6)	(42.9)		
Male	9	69	96	723	0.129	
	(40.9)	(47.9)	(43.1)	(42.5)		
Female	13	45	80	550	**0.010**	
	(72.2)	(51.1)	(42.1)	(43.4)		
Lymphopenia‖	14	62	138	796	0.086	0.679
	(32.6)	(25.1)	(30.7)	(25.3)		
Male	6	42	71	453	0.425	
	(27.3)	(26.6)	(29.3)	(24.8)		
Female	8	20	67	343	0.146	
	(38.1)	(22.5)	(32.4)	(25.9)		
C-reactive protein (mg/L), median, [IQR]	44.3 [8.1–176.4]	14.8 [3.4–44.9]	27.8 [7.2–95.7]	12.9 [3.3–39.0]	**<0.001**	0.731
Male	44.3 [7.8–176.4]	14.6 [4.5–39.5]	22.6 [5.2–82.9]	12.5 [3.0–37.8]	**<0.001**	
Female	43.5 [8.5–130.7]	15.7 [2.4–53.6]	34.4 [11.9–107.6]	13.1 [3.6–41.3]	**<0.001**	
Underlying condition**	9	61	129	803	0.637	0.291
	(20.9)	(24.7)	(28.7)	(25.5)		
Male	7	35	65	429	0.713	
	(31.8)	(22.2)	(26.9)	(23.5)		
Female	2	26	64	374	0.988	
	(9.5)	(29.2)	(30.9)	(28.3)		

* Overall *P* value obtained from multinomial logistic regression adjusted for age and site (where applicable). *P* values comparing co-detection groups are presented for males and females together, followed by sex-stratified *P* values listed below the grouped *P* values. Bold values denote statistical significance at the *P* < 0.05 level.

† Effect modification by sex, as indicated by interaction term *P* < 0.05 adjusted for site and age.

‡ Excludes South Africa due to near uniformity of receiving oxygen at South Africa.

§ Restricted to children 6 months of age or older.

¶ Severe acute malnutrition: weight-for-height Z-score <-3 SD or middle arm circumference Z-score <−3 SDs or diagnosis of acute severe malnutrition.

‖ Below 3000 cells per microliter of blood (3 × 10^9^/L).

** Underlying conditions: cerebral palsy, congenital heart disease/defect, congenital abnormalities, developmental delay, severe malnutrition, prematurity in an infant <6 months old. The number of days with cough, fever, difficulty breathing, wheeze or runny nose, whichever symptom is longest.

CoV indicates coronavirus; HDSpn, high-density *streptococcus pneumoniae*; IQR, interquartile range; NP/OP, nasopharyngeal/oropharyngeal; WHO, World Health Organization.

**FIGURE 1.**
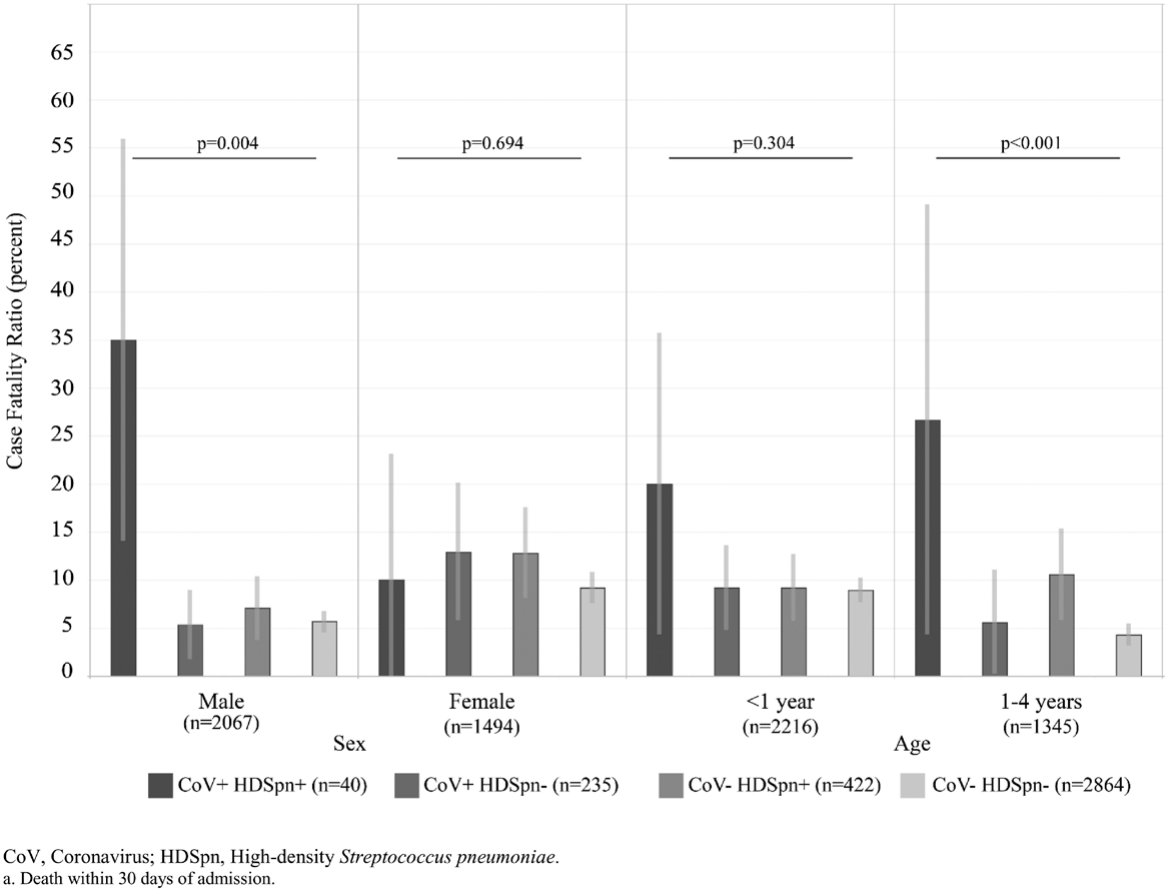
Case fatality ratio (death within 30 days of admission) among children hospitalized with severe or very severe pneumonia, grouped by human endemic Cov and HDSpn nasopharyngeal/oropharyngeal co-detection and stratified by sex.

The overall case fatality ratio (CFR) among all PERCH HIV-negative cases was higher in females (8.9%) than males (7.4%, *P* < 0.001) (Supplemental Digital Content 5, http://links.lww.com/INF/E363). The CFR was highest in CoV+/HDSpn+ cases (22.5%, n = 9/40) compared with the other coinfection groups (7.2%–9.7%, *P* = 0.053) (Supplemental Digital Content 6, http://links.lww.com/INF/E363). When stratified by sex, this association was seen only among males (interaction by sex *P* = 0.02) among whom CoV+/HDSpn+ CFR was 35.0% (n = 7/20, 95% CI 18.1%–56.7%) compared with 5.3%–7.1% in the other groups (*P* = 0.004). In females, the CFR among CoV+/HDSpn+ was 10.0% (n = 2/20) and 9.2%–12.9% in other groups (*P* = 0.69, Fig. 1). After adjusting for age, site and malnutrition, the odds ratio of mortality in CoV+/HDSpn+ male cases compared with CoV+/HDSpn−, CoV−/HDSpn+ and CoV−/HDSpn− male cases was 11.6 (95% CI 3.1–44.4), 5.9 (1.7–20.3) and 8.6 (3.1–24.2), respectively (Supplemental Digital Content 7, http://links.lww.com/INF/E363). Among male children with CoV who died, 46.7% (n = 7/15; 95% CI 21.4%–71.9%) were HDSpn+ in NP/OP. Males without pneumococcus detected on NP/OP swabs had significantly higher CFR than males with low-density (<6.9 log_10_ copies/mL) pneumococcal upper respiratory tract carriage (CoV+: 10.2% versus 3.0%, *P* = 0.05; CoV−: 7.4% versus 4.9%, *P* = 0.02) Supplemental Digital Content 8, http://links.lww.com/INF/E363). CFR was similar across pneumococcal density categories for female children (range 8.2%–13.3%).

The CoV NP/OP viral load was similar among males and females (median 5.2 and 5.3 log_10_ copies/mL, respectively) and did not differ significantly by pneumococcal load (range 4.9–5.8 log_10_ copies/mL between noncarriers, LDSpn and HDSpn, *P* = 0.36) (Supplemental Digital Content 9 and 10, http://links.lww.com/INF/E363). Viral load was slightly higher among children who died compared with those that survived (median 5.7 versus 5.2 log_10_ copies/mL; *P* = 0.09), and was significantly higher when restricted to HDSpn+ cases (median 7.2 versus 5.6 log_10_ copies/mL, *P* = 0.03). Pneumococcal and CoV NP/OP densities and other organisms detected from sterile site and NP/OP swabs are described for fatal CoV+ cases with and without HDSpn co-detection in Supplemental Digital Content 11 and 12, http://links.lww.com/INF/E363. Among CoV+/HDSpn+ children who died, a median of 2 additional organisms were detected by NP/OP PCR (Supplemental Digital Content 13, http://links.lww.com/INF/E363), and 2 nonpneumococcal organisms were detected from sterile sites.

Two sensitivity analyses were conducted that evaluated other definitions of CoV and HDSpn: (1) included CoV detected in induced sputum specimens, which added 97 CoV+ cases, and included microbiologically confirmed pneumococcal pneumonia cases to HDSpn+, which added three cases; (2) decreased the pneumococcal density threshold to 6.6 log_10_ copies/mL, which added 215 HDSpn+ cases (Supplemental Digital Content 14, http://links.lww.com/INF/E363). All findings were consistent with the primary analysis.

### Co-detection of Other Potential Pathogens

High-density *H. influenzae* colonization of the upper airway was the strongest bacterial predictor of CoV detection by random forest analysis (data not shown), but co-detection of CoV and high-density *H. influenzae* was not associated with mortality (CFR: 9.5% versus 6.7%–10.5% in other groups) (Supplemental Digital Content 15, http://links.lww.com/INF/E363). Similarly, mortality was not higher relative to other groups in cases where both CoV and *S. aureus* were detected in the NP/OP, or with co-detection of HDSpn and influenza A/B/C, human metapneumovirus, Para 1/3 or RSV A/B. Although influenza was rarely detected during PERCH, there were no deaths among the 22 HDSpn+/influenza+ co-detected cases. There were no differences by sex for any combination of pathogens except HDSpn and Para 1/3 where co-detection had a higher CFR among females (31.3% versus 7.6%–11.1%, *P* = 0.03) but not males (8.0% versus 2.6%–9.5%, *P* = 0.05).

## DISCUSSION

Prevalence of endemic coronavirus species detected in the upper respiratory tract of children <5 years hospitalized with severe or very severe pneumonia in pre-COVID-19 years was 7.5%, which was lower than prevalence in age-matched community controls without pneumonia (10.0%). Co-detection of human endemic CoV species and HDSpn, a marker of pneumococcal pneumonia, was infrequent (1.1%), but in male children only was associated with higher case fatality and more severe disease compared with detection of CoV or *S. pneumoniae* alone. Case fatality was 35.0% in co-infected males compared with 5.3%–7.1% in the other infection combinations, whereas, in females, the case fatality was 10.0% versus 9.2%–12.9%, respectively. High-density pneumococcus was detected in 12.6% of cases overall, 14.8% among those with CoV detected and 18.2% of cases that died with no differences by sex, but high-density pneumococcus was detected in 47% (n = 7) of the 15 male children that died who had endemic CoV detected.

Endemic CoV species were not reported to be an important cause of severe pneumonia in the PERCH study because detection was low in cases and higher in controls.^35^ The more complex evaluation of pathogen and sex interaction presented here identified a subset of CoV-infected children with severe disease and fatal outcomes. Co-pathogenesis in pneumonia involves complex interactions between pathogens and host. Respiratory viruses may disrupt the lung physiology and generate immunopathologies that promote subsequent bacterial infection.^43^ Bacterial infections can increase morbidity of viral infections by increasing viral load and decreasing clearance.^1,44^ Among children with NP/OP co-detection of CoV and high-density pneumococcus, those that died had significantly higher CoV viral loads than those that survived (Supplemental Digital Content 9, http://links.lww.com/INF/E363). However, viral loads were similar between males and females among those with co-detection (Supplemental Digital Content 10, http://links.lww.com/INF/E363), so high viral load alone may not explain higher mortality in males. Certain pathogens inhibit the host immune response and increase susceptibility to secondary infections.^45,46^ There is evidence that CoV-NL63 strongly enhances streptococcal adherence to epithelial cells in human airway epithelium cultures and conversely does not affect adhesion of *S. aureus*, *H. influenzae* or *Pseudomonas aeruginosa*, which aligns with our findings of co-detection with these other pathogens.^33^

NP/OP pneumococcal carriage itself, and not solely superinfection in the lower respiratory tract, may play a role in severity. Virulence factors associated with nasopharyngeal colonization and biofilm formation are associated with lower respiratory tract adhesion, development of pneumonia, invasion, inflammation and cytotoxicity.^47^ High pneumococcal nasopharyngeal density also primes alveolar macrophages and leads to increased responsiveness to pneumococcus and other pathogens.^48–50^ High-density pneumococcal carriage in the upper respiratory tract may be a marker of microbiome dysbiosis, and pneumococcus may play a role in a wider relationship between the respiratory tract microbiome and severity. Studies have suggested that low-density pneumococcal carriage in adults is associated with fewer microbiome perturbations, lower rates of viral coinfection and replication and decreased mucosal cytokine responses when compared with high-density carriage or noncarriage.^22^ This is consistent with our findings of highest mortality in children with noncarriage of pneumococcus and high-density pneumococcal carriage, particularly with CoV detection among male children (Supplemental Digital Content 8, http://links.lww.com/INF/E363).^22,51^ The microbiome has sex-dependent effects on immune function and priming, and males have higher absolute abundance of bacteria in the upper respiratory tract, which could contribute to observed differences by sex.^52,53^

In most developing country settings, female children have lower mortality rates than males due to biologic advantages, unless females have lower access to care or other disadvantages.^54^ In the context of COVID-19 in adults, males have generally constituted a higher proportion of hospitalized COVID-19 cases and had higher case fatality.^30–32,55^ IPD is also known to affect males disproportionately.^26–29^ Behavioral and immunologic factors are likely to contribute some of the differential severity by sex.^32,56^ However, immunologic differences may be less pronounced in children, and in the PERCH study, cases were more likely to be male and case fatality was higher in females (8.9% versus 7.4%) suggesting possible greater care-seeking for males in this study population. This suggests that external biologic factors may play a role in explaining the excess deaths observed in male children in the PERCH study and our results warrant consideration of the potential role of *S. pneumoniae* in differential severity by sex.

There are important limitations to this analysis. Although this was a large study with almost 4000 cases and 274 deaths with evaluable data, the analysis required multiple stratifications that resulted in a small sample size of the key subgroup of interest, that of males and females with co-detection of CoV and high-density pneumococcus. As a result, we were unable to evaluate outcomes by endemic CoV subtypes (Supplemental Digital Content 16, http://links.lww.com/INF/E363). Although the investigation was hypothesis-driven and the PERCH study was designed to evaluate causes and severity of pneumonia, this was not a prespecified analysis of the main study. Therefore, results shown here could be incidental and should be confirmed in other studies. There was higher overall mortality among female children in PERCH, suggesting potential conservative bias in estimates of sex differences. We used high-density pneumococcal detection in the NP/OP as a marker of pneumococcal pneumonia, but it is not a confirmatory measure as it has poor specificity,^20^ and sensitivity is reduced by prior exposure to antibiotics,^57^ which was common at PERCH sites. Furthermore, detection of organisms in the upper respiratory tract may not be a reliable surrogate for lower respiratory tract infection. Most cases and controls in PERCH had four or more pathogens detected on NP/OP, including the cases who died with CoV and high-density pneumococcus detected, making it difficult to attribute causation for any specific pneumonia case.^35^ One had *S. aureus* detected in pleural fluid and in PERCH was attributed at the cause of the pneumonia, but most of the additional organisms detected in the COV+/high-density pneumococcus deaths were also commonly found in controls without pneumonia.

A further limitation was our inability to fully explore the effect of malnutrition on participant outcomes in this analysis. Results adjusted for chronic malnutrition were consistent with overall findings, but because of the small sample size may not have adequately accounted for all factors that may have contributed to the higher mortality in males. Although effect of sex was not statistically significant, males with co-detection were more likely to have height-for-age Z-score < −3 SDs (30.0%) compared with females (11.1%). Markers of severe acute malnutrition were statistically different between males and females, but this may indicate severity of illness as vomiting, diarrhea and systemic involvement were more prevalent in the co-detection group and in children that died. Nonetheless, malnutrition associated with pediatric pneumonia should be recognized as an important risk factor for mortality.^58^ None of the children with co-detection who died had underlying conditions other than severe malnutrition. The similar prevalence of co-detection in community controls suggests that human endemic CoV species may not be a sufficient etiologic cause of pneumonia, alone or in combination with pneumococcus, but may interact with pneumococcus to exacerbate disease under specific conditions yet to be determined. Sensitivity analyses that increased sample size slightly were consistent with our primary analysis.

Any extensions from human endemic CoV species to COVID-19 may be inappropriate because epidemiologic and clinical manifestations of SARS-CoV-2 are different from endemic CoV species and findings from a pediatric population may not be relevant to adults as children have lower severity of COVID-19 compared with adults, possibly due to lower ACE2 receptor expression.^59^ Nonetheless, an analysis of adults in England’s national surveillance system reported coinfection of IPD and COVID-19 being rare, but associated with a significant 7.8-fold increase in the case fatality rate.^16^ Carriage and IPD due to vaccine-type pneumococci can be reduced by pneumococcal vaccination,^60^ and recent reports have suggested a potential inverse association between pneumococcal vaccination and both endemic CoV and SARS-CoV-2 infection.^34,61,62^ However, pneumococcal conjugate vaccine vaccination status was not associated with co-detection in our study.

*S. pneumoniae* co-pathogenesis may contribute to increased morbidity and mortality from CoV infection among children with pneumonia. Coronavirus and HDSpn co-detection was rare, but HDSpn was present in almost a quarter of CoV-positive very severe cases, and in nearly half of CoV-positive males who died. Further studies are needed to confirm these findings, and to elucidate the role of high-density pneumococcal carriage in the upper respiratory tract on immunologic priming, microbiome dysbiosis and other biologic mechanisms of exacerbation. Further efforts to detect pneumococcal coinfection with endemic coronaviruses and SARS-CoV-2 may be warranted, along with potential evaluations of pneumococcal vaccination and colonization density as predictors of disease progression.

## ACKNOWLEDGMENTS

We recognize the support provided by the Institutional Review Boards for study oversight. We appreciate the helpful discussions with Scott Zeger and our many colleagues. Finally, we gratefully recognize the parents and children who participated in this study and express our gratitude for their commitment to the advancement of knowledge toward better health for children in and beyond their community.

## Supplementary Material


