# Supplementary material for: Upper Respiratory Tract Co-detection of Human Endemic Coronaviruses and High-density Pneumococcus Associated With Increased Severity Among HIV-Uninfected Children Under 5 Years Old in the PERCH Study
Source: Pediatr Infect Dis J. 2021 Apr 19;40(6):503–12. doi: 10.1097/INF.0000000000003139 (PMC8104011; doi:10.1097/INF.0000000000003139)
Supplement: Supplementary file 1 [file inf-40-0503-s001.docx]

Supplemental Tables: Upper respiratory tract co-detection of human endemic coronaviruses and high-density pneumococcus associated with increased severity among HIV-uninfected children under 5 years old in the PERCH study

**Contents**

[1. Distribution of human endemic coronavirus (CoV) and high-density pneumococcus (HDSpn) co-detection category by PERCH case-control status and sex 3](#_Toc65938886)

[2. Distribution of human endemic coronavirus (CoV) and high-density pneumococcus (HDSpn) co-detection category by PERCH case-control status and age 4](#_Toc65938887)

[3. Clinical Characteristics of Children Hospitalized with Severe or Very Severe Pneumonia by NP/OP Co-detection Status of Endemic Coronavirus (CoV-NL63, CoV-229E, CoV-OC43 or CoV-HKU1) and High-density S. pneumoniae^a^, by sex. 5](#_Toc65938888)

[4. C-Reactive protein levels by human endemic coronavirus (CoV) and high-density pneumococcus (HDSpn) NP/OP co-detection status and sex 9](#_Toc65938889)

[5. Overall mortality by sex among PERCH cases 10](#_Toc65938890)

[6. Mortality by age and sex category by human endemic coronavirus (CoV) and high-density pneumococcus (HDSpn) NP/OP co-detection category 11](#_Toc65938891)

[7. Adjusted Odds Ratios for Morbidity and Mortality for Nasopharyngeal/Oropharyngeal Co-Detection with Human Endemic Coronavirus (Cov) and High-Density *S. Pneumoniae* (HDSpn) among Children Hospitalized with Severe or Very Severe Pneumonia, Adjusted for Age, Site Region, and Malnutrition. 12](#_Toc65938892)

[8a. Case fatality ratio by NP/OP co-detection of any density *S. pneumoniae* (Spn) with human endemic coronavirus (CoV) 13](#_Toc65938893)

[8b. Case fatality ratio by NP/OP co-detection of high (HDSpn) or low (LDSpn) density *S. pneumoniae* with coronavirus (CoV) 13](#_Toc65938894)

[9. Coronavirus (CoV) density (log_10_ copies/mL) by sex, mortality, and *S. pneumoniae* (Spn) co-detection status 14](#_Toc65938895)

[10. Coronavirus (CoV) viral load by high-density *S. pneumoniae* (HDSpn) co-detection status and sex 15](#_Toc65938896)

[11. Clinical and etiologic findings among cases who died with human endemic coronavirus (CoV) in NP/OP 16](#_Toc65938897)

[11a. With high-density *S. pneumoniae* (HDSpn) NP/OP co-detection 16](#_Toc65938898)

[11b. Without high-density *S. pneumoniae* (HDSpn) NP/OP co-detection 17](#_Toc65938899)

[12. High-density pneumococcus (HDSpn) detection and pneumococcal load in clinical samples by co-detection category with human endemic coronavirus (CoV) 19](#_Toc65938900)

[13. Median number of pathogens positive on NP/OP PCR by human endemic coronavirus (CoV) and high-density pneumococcus (HDSpn) co-detection category, among cases 20](#_Toc65938901)

[14. Sensitivity analyses evaluating association of clinical outcomes by co-detection group including additional CoV and Spn cases 21](#_Toc65938902)

[14a. Adding microbiologically confirmed pneumococcal pneumonia^a^ to high density *S. pneumoniae* (HDSpn) and adding human endemic CoV detected in induced sputum to cases with CoV detected in NP/OP 21](#_Toc65938903)

[14b. Lowering the threshold to >6.6 log10 copies/mL to define high density *S. pneumoniae* (HDSpn) 22](#_Toc65938904)

[15. Case fatality risk by NP/OP co-detection of other pathogen combinations, by sex 23](#_Toc65938905)

[15a. High-density *H. influenzae* (HDHinf) and any coronavirus (CoV) 23](#_Toc65938906)

[15b. *S. aureus* (Saur) and any coronavirus (CoV) 23](#_Toc65938907)

[15c. High-density *S. pneumoniae* (HDSpn) and any influenza A, B or C (Flu) 23](#_Toc65938908)

[15d. High-density *S. pneumoniae* (HDSpn) and HMPV 23](#_Toc65938909)

[15e. High-density *S.pneumoniae* (HDSpn) and RSV A/B 24](#_Toc65938910)

[15f. High-density *S.pneumoniae* (HDSpn) and Parainfluenza 1 or 3 (Para1/3) 24](#_Toc65938911)

[16. Mortality by coronavirus species and gender by human endemic coronavirus (CoV) and high-density pneumococcus (HDSpn) NP/OP co-detection category 25](#_Toc65938912)

## 1. Distribution of human endemic coronavirus (CoV) and high-density pneumococcus (HDSpn) co-detection category by PERCH case-control status and sex

| **Case / Control** | **Co-detection Category** | | | |
| --- | --- | --- | --- | --- |
|  | **A. CoV+ / HDSpn+** | **B. CoV+ / HDSpn-** | **C. CoV- / HDSpn+** | **D. CoV- and HDSpn -** |
|  | **n=90** | **n=701** | **n=781** | **n=7292** |
| **Male** |  |  |  |  |
| **Case** n = 2248  row%  col% | 22 | 158 | 242 | 1826 |
|  | 1.0 | 7.0 | 10.8 | 81.2 |
|  | 46.8 | 41.3 | 61.1 | 46.5 |
| **Control** n = 2505  row%  col% | 25 | 225 | 154 | 2101 |
|  | 1.0 | 9.0 | 6.2 | 83.9 |
|  | 53.2 | 58.8 | 38.9 | 53.5 |
| **Female** |  |  |  |  |
| **Case** n=1640  row%  col% | 21 | 89 | 207 | 1323 |
|  | 1.3 | 5.4 | 12.6 | 80.7 |
|  | 48.8 | 28.1 | 53.8 | 39.3 |
| **Control** n=2470  row%  col% | 22 | 228 | 178 | 2042 |
|  | 0.9 | 9.2 | 7.2 | 82.7 |
|  | 51.2 | 71.9 | 46.2 | 60.7 |

## 2. Distribution of human endemic coronavirus (CoV) and high-density pneumococcus (HDSpn) co-detection category by PERCH case-control status and age

| **Case / Control** | **Co-detection Category** | | | |
| --- | --- | --- | --- | --- |
|  | **A. CoV+ / HDSpn+** | **B. CoV+ / HDSpn-** | **C. CoV- / HDSpn+** | **D. CoV- and HDSpn -** |
|  | **n=90** | **n=701** | **n=781** | **n=7292** |
| **Case** |  |  |  |  |
| **1-5m (n=1581)** | 17 | 116 | 177 | 1271 |
| Row % | 1.1 | 7.3 | 11.2 | 80.4 |
| Col % | 39.5 | 47.0 | 39.4 | 40.4 |
| **6-11m (n=884)** | 10 | 58 | 107 | 709 |
| Row % | 1.1 | 6.6 | 12.1 | 80.2 |
| Col % | 23.3 | 23.5 | 23.8 | 22.5 |
| **12-23m (n=883)** | 8 | 45 | 119 | 711 |
| Row % | 0.9 | 5.1 | 13.5 | 80.5 |
| Col % | 18.6 | 18.2 | 26.5 | 22.6 |
| **24-59m (n=540)** | 8 | 28 | 46 | 458 |
| Row % | 1.5 | 5.2 | 8.5 | 84.8 |
| Col % | 18.6 | 11.3 | 10.2 | 14.5 |
| **Control** |  |  |  |  |
| **1-5m (n=1552)** | 17 | 143 | 117 | 1275 |
| Row % | 1.1 | 9.2 | 7.5 | 82.2 |
| Col % | 36.2 | 31.5 | 35.2 | 30.8 |
| **6-11m (n=1182)** | 10 | 116 | 74 | 982 |
| Row % | 0.9 | 9.8 | 6.3 | 83.1 |
| Col % | 21.3 | 25.6 | 22.3 | 23.7 |
| **12-23m (n=1232)** | 14 | 106 | 70 | 1042 |
| Row % | 1.1 | 8.6 | 5.7 | 84.6 |
| Col % | 29.8 | 23.4 | 21.1 | 25.2 |
| **24-59m (n=1010)** | 6 | 89 | 71 | 844 |
| Row % | 0.6 | 8.8 | 7.0 | 83.6 |
| Col % | 12.8 | 19.6 | 21.4 | 20.4 |

Chi-square p-value for difference among cases p=0.077, among controls p=0.540

## 3. Clinical Characteristics of Children Hospitalized with Severe or Very Severe Pneumonia by NP/OP Co-detection Status of Endemic Coronavirus (CoV-NL63, CoV-229E, CoV-OC43 or CoV-HKU1) and High-density S. pneumoniae^a^, by sex.

| **Characteristics** | **No. (% with available information)** | | | |  | **Adjusted p-value^a^** | | |
| --- | --- | --- | --- | --- | --- | --- | --- | --- |
|  | **A. CoV+ / HDSpn+**  **n=43** | **B. CoV+ / HDSpn-**  **n=247** | **C. CoV- / HDSpn+**  **n=449** | **D. CoV- or HDSpn -**  **n=3149** | **Overall** | **A vs B** | **A vs C** | **A vs D** |
| **Very severe pneumonia (2005 WHO definition)** | 23 | 81 | 174 | 969 | **<0.001** | **0.013** | 0.084 | **0.004** |
|  | (53.5) | (32.8) | (38.8) | (30.8) |  |  |  |  |
| Male | 13/22 | 46/158 | 84/242 | 539/1826 | **0.039** | **0.021** | 0.092 | **0.015** |
|  | (59.1) | (29.1) | (34.7) | (29.5) |  |  |  |  |
| Female | 10/21 | 35/89 | 90/207 | 430/1323 | **0.009** | 0.324 | 0.623 | 0.131 |
|  | (47.6) | (39.3) | (43.5) | (32.5) |  |  |  |  |
| **Hypoxemia at admission** | 17 | 90 | 183 | 1098 | 0.061 | 0.631 | 0.853 | 0.503 |
|  | (39.5) | (36.6) | (40.9) | (35.0) |  |  |  |  |
| Male | 8 | 49 | 91 | 592 | 0.182 | 0.735 | 0.740 | 0.716 |
|  | (36.4) | (31.0) | (37.6) | (32.5) |  |  |  |  |
| Female | 9 | 41 | 92 | 506 | 0.265 | 0.900 | 0.796 | 0.644 |
|  | (42.9) | (46.6) | (44.7) | (38.3) |  |  |  |  |
| **WHO defined very severe or hypoxemia** | 30 | 130 | 254 | 1601 | **0.002** | **0.026** | 0.070 | **0.008** |
|  | (69.8) | (52.9) | (56.6) | (50.9) |  |  |  |  |
| Male | 16 | 74 | 125 | 885 | 0.094 | **0.042** | 0.103 | **0.033** |
|  | (72.7) | (46.8) | (51.7) | (48.5) |  |  |  |  |
| Female | 14 | 56 | 129 | 716 | **0.015** | 0.400 | 0.371 | 0.125 |
|  | (66.7) | (63.6) | (62.3) | (54.2) |  |  |  |  |
| **Supplemental oxygen (ever)^b,c^** | 13/36 | 48/192 | 99/367 | 687/2475 | **0.001** | **0.030** | 0.179 | **0.013** |
|  | (36.11) | (25.0) | (27.0) | (27.8) |  |  |  |  |
| Male | 9/20 | 26/126 | 59/206 | 373/1460 | **<0.001** | **0.010** | 0.331 | **0.012** |
|  | (45.0) | (20.6) | (28.6) | (25.5) |  |  |  |  |
| Female | 4/16 | 22/66 | 40/161 | 314/1015 | 0.584 | 0.834 | 0.348 | 0.397 |
|  | (25.0) | (33.3) | (24.8) | (30.9) |  |  |  |  |
| **Tachypnea^c^** | 38 | 199 | 398 | 2546 | **0.044** | 0.528 | 0.858 | 0.479 |
|  | (88.4) | (81.2) | (88.6) | (81.5) |  |  |  |  |
| Male | 17 | 122 | 220 | 1479 | **0.009** | 0.900 | **0.085** | 0.418 |
|  | (77.3) | (77.2) | (90.9) | (81.5) |  |  |  |  |
| Female | 21 | 77 | 178 | 1067 | 0.423 | 0.948 | 0.864 | 0.910 |
|  | (100.0) | (88.5) | (86.0) | (81.5) |  |  |  |  |
| **Fever** | 37 | 193 | 400 | 2533 | **0.001** | 0.311 | 0.389 | 0.593 |
|  | (86.1) | (78.1) | (89.1) | (80.4) |  |  |  |  |
| Male | 20 | 121 | 212 | 1462 | **0.025** | 0.200 | 0.892 | 0.367 |
|  | (90.9) | (76.6) | (87.6) | (80.1) |  |  |  |  |
| Female | 17 | 72 | 188 | 1071 | **0.017** | 0.855 | 0.199 | 0.871 |
|  | (81.0) | (80.9) | (90.8) | (81.0) |  |  |  |  |
| **Observed cough** | 22 | 168 | 302 | 2215 | 0.175 | 0.174 | 0.078 | **0.030** |
|  | (51.2) | (68.0) | (67.4) | (70.6) |  |  |  |  |
| Male | 10 | 110 | 167 | 1310 | 0.204 | 0.193 | 0.089 | **0.031** |
|  | (45.5) | (69.6) | (69.0) | (71.9) |  |  |  |  |
| Female | 12 | 58 | 135 | 905 | 0.720 | 0.681 | 0.354 | 0.395 |
|  | (57.1) | (65.2) | (65.5) | (68.7) |  |  |  |  |
| **Vomiting** | 12 | 49 | 104 | 643 | 0.382 | 0.404 | 0.560 | 0.252 |
|  | (28.6) | (19.8) | (23.2) | (20.4) |  |  |  |  |
| Male | 9 | 28 | 52 | 358 | 0.133 | **0.040** | 0.076 | **0.029** |
|  | (40.9) | (17.7) | (21.5) | (19.6) |  |  |  |  |
| Female | 3 | 21 | 52 | 285 | 0.726 | 0.254 | 0.318 | 0.448 |
|  | (15.0) | (23.6) | (25.1) | (21.5) |  |  |  |  |
| **Diarrhea** | 9 | 38 | 86 | 439 | 0.062 | 0.219 | 0.975 | 0.313 |
|  | (20.9) | (15.4) | (19.2) | (13.9) |  |  |  |  |
| Male | 5 | 27 | 53 | 248 | **0.004** | 0.481 | 0.605 | 0.326 |
|  | (22.7) | (17.1) | (22.0) | (13.6) |  |  |  |  |
| Female | 4 | 11 | 33 | 191 | 0.816 | 0.283 | 0.760 | 0.637 |
|  | (19.1) | (12.4) | (16.0) | (14.4) |  |  |  |  |
| **Abnormal chest X-ray ^c^** | 15/31 | 110/213 | 220/377 | 1381/2706 | 0.077 | 0.834 | 0.294 | 0.930 |
|  | (48.4) | (51.6) | (58.4) | (51.0) |  |  |  |  |
| Male | 11/17 | 60/137 | 119/205 | 783/1585 | **0.030** | 0.081 | 0.664 | 0.215 |
|  | (64.7) | (43.8) | (58.1) | 49.4) |  |  |  |  |
| Female | 4/14 | 50/76 | 101/172 | 598/1121 | **0.030** | **0.025** | **0.023** | 0.127 |
|  | (28.6) | (65.8) | (58.7) | (53.4) |  |  |  |  |
| **Weight-for-height Z-score < -3 SDs** | 5 | 25 | 73 | 338 | 0.118 | 0.904 | 0.282 | 0.852 |
|  | (11.9) | (10.6) | (16.6) | (11.1) |  |  |  |  |
| Male | 4 | 19 | 43 | 184 | 0.052 | 0.852 | 0.901 | 0.333 |
|  | (19.1) | (12.3) | (18.1) | (10.4) |  |  |  |  |
| Female | 1 | 6 | 30 | 154 | 0.247 | 0.771 | 0.177 | 0.249 |
|  | (4.8) | (7.3) | (14.9) | (12.1) |  |  |  |  |
| **Weight-for-age Z-score < -3 SDs ^c^** | 7 | 31 | 84 | 491 | 0.216 | 0.575 | 0.677 | 0.874 |
|  | (16.7) | (12.7) | (18.7) | (15.7) |  |  |  |  |
| Male | 6 | 18 | 54 | 280 | **0.014** | **0.046** | 0.705 | 0.126 |
|  | (28.6) | (11.5) | (22.3) | (15.4) |  |  |  |  |
| Female | 1 | 13 | 30 | 212 | 0.604 | 0.173 | 0.348 | 0.203 |
|  | (4.8) | (14.8) | (14.5) | (16.1) |  |  |  |  |
| **Arm circumference for age Z-score < -3 SDs^d^** | 4 | 6 | 34 | 120 | 0.172 | 0.237 | 0.930 | 0.528 |
|  | (15.4) | (4.6) | (12.8) | (6.5) |  |  |  |  |
| Male | 3 | 2 | 21 | 67 | **0.005** | **0.043** | 0.546 | 0.121 |
|  | (21.4) | (2.6) | (14.6) | (6.4) |  |  |  |  |
| Female | 1 | 4 | 13 | 53 | 0.828 | 0.447 | 0.577 | 0.429 |
|  | (8.3) | (7.4) | (10.7) | (6.8) |  |  |  |  |
| **Severe acute malnutrition^e^** | 7 | 43 | 97 | 556 | 0.635 | 0.714 | 0.257 | 0.473 |
|  | (16.3) | (17.7) | (21.8) | (17.8) |  |  |  |  |
| Male | 6 | 26 | 55 | 295 | 0.298 | 0.480 | 0.928 | 0.308 |
|  | (27.3) | (16.6) | (22.9) | (16.3) |  |  |  |  |
| Female | 1 | 17 | 42 | 261 | 0.308 | 0.149 | 0.095 | 0.075 |
|  | (4.8) | (19.8) | (20.5) | (20.0) |  |  |  |  |
| **Height-for-age Z-score < -3 SDs** | 8 | 29 | 67 | 522 | 0.192 | 0.062 | 0.421 | 0.268 |
|  | (21.1) | (14.2) | (18.1) | (19.8) |  |  |  |  |
| Male | 6 | 21 | 37 | 329 | 0.234 | 0.053 | 0.305 | 0.160 |
|  | (30.0) | (15.7) | (18.9) | (21.5) |  |  |  |  |
| Female | 2 | 8 | 30 | 193 | 0.427 | 0.491 | 0.952 | 0.972 |
|  | (11.1) | (11.4) | (17.1) | (17.4) |  |  |  |  |
| **Leukocytosis** | 22 | 114 | 176 | 1273 | **0.004** | 0.517 | 0.098 | **0.018** |
|  | (55.0) | (49.1) | (42.6) | (42.9) |  |  |  |  |
| Male | 9 | 69 | 96 | 723 | 0.129 | 0.606 | 0.930 | 0.730 |
|  | (40.9) | (47.9) | (43.1) | (42.5) |  |  |  |  |
| Female | 13 | 45 | 80 | 550 | **0.010** | 0.165 | **0.011** | **0.003** |
|  | (72.2) | (51.1) | (42.1) | (43.4) |  |  |  |  |
| **Lymphopenia**^f^ | 14 | 62 | 138 | 796 | 0.086 | 0.228 | 0.944 | 0.370 |
|  | (32.6) | (25.1) | (30.7) | (25.3) |  |  |  |  |
| Male | 6 | 42 | 71 | 453 | 0.425 | 0.856 | 0.705 | 0.935 |
|  | (27.3) | (26.6) | (29.3) | (24.8) |  |  |  |  |
| Female | 8 | 20 | 67 | 343 | 0.146 | 0.065 | 0.611 | 0.230 |
|  | (38.1) | (22.5) | (32.4) | (25.9) |  |  |  |  |
| **C-reactive protein (mg/L), median, [IQR]** | 44.3  [8.1 - 176.4] | 14.8  [3.4 - 44.9] | 27.8 [7.2 - 95.7] | 12.9 [3.3 - 39.0] | **<0.001** | **<0.001** | 0.094 | **<0.001** |
| Male | 44.3 [7.8 - 176.4] | 14.6 [4.5 - 39.5] | 22.6 [5.2 - 82.9] | 12.5 [3.0 - 37.8] | **<0.001** | **<0.001** | **0.015** | **<0.001** |
| Female | 43.5 [8.5 - 130.7] | 15.7 [2.4 - 53.6] | 34.4 [11.9 - 107.6] | 13.1 [3.6 - 41.3] | **<0.001** | **0.029** | 0.967 | **0.004** |
| **Underlying condition**^g^ | 9 | 61 | 129 | 803 | 0.637 | 0.371 | 0.294 | 0.214 |
|  | (20.9) | (24.7) | (28.7) | (25.5) |  |  |  |  |
| Male | 7 | 35 | 65 | 429 | 0.713 | 0.968 | 0.806 | 0.620 |
|  | (31.8) | (22.2) | (26.9) | (23.5) |  |  |  |  |
| Female | 2 | 26 | 64 | 374 | 0.988 | 0.221 | 0.212 | 0.209 |
|  | (9.5) | (29.2) | (30.9) | (28.3) |  |  |  |  |

WHO, World Health Organization; SD, Standard deviation; IQR, Inter-quartile range; CoV, Coronavirus; HDSpn, High-density *Streptococcus pneumoniae*; NP/OP, Nasopharyngeal/oropharyngeal.

a. P-values for pairwise comparisons of categorical variables obtained from logistic regression model adjusted for age and site (where applicable). P-values comparing co-detection groups are presented for males and females together, followed by sex-stratified p-values listed below the grouped p-values.

b. Excludes South Africa due to near uniformity of receiving oxygen at the South Africa PERCH site.

c. Significant effect modification by sex, as indicated by interaction term p<0.05 adjusted for site and age.

d. Restricted to children aged 6 months or older.

e. Severe acute malnutrition: weight-for-height Z-score <-3 SD or middle arm circumference Z-score <-3 SDs or diagnosis of acute severe malnutrition.

f. Below 3,000 cells per microliter of blood (3 × 10^9^ per liter)

g. Underlying conditions: cerebral palsy, congenital heart disease/defect, congenital abnormalities, developmental delay, severe malnutrition, prematurity in an infant <6 months old. f. The number of days with cough, fever, difficulty breathing, wheeze, or runny nose, whichever symptom is longest.

## 4. C-Reactive protein levels by human endemic coronavirus (CoV) and high-density pneumococcus (HDSpn) NP/OP co-detection status and sex


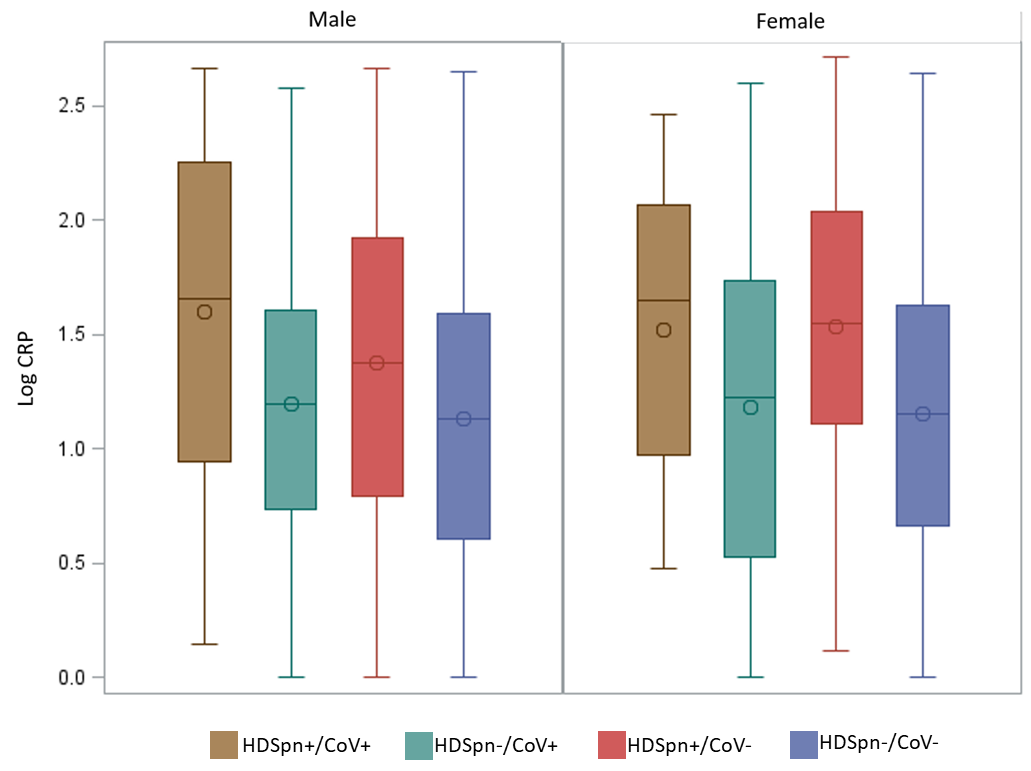


Kruskal-Wallis test p < 0.001 for differences in log CRP among males and also among females. Median values indicated by horizontal line. Mean values indicated by circles. Quartile 1 and quartile 3 indicated by the bottom and top of box, respectively.

## 5. Overall mortality by sex among PERCH cases

| **Sex of Child** | **Died** | | **aOR**  **(95% CI)** | **p-value** |
| --- | --- | --- | --- | --- |
|  | **Yes** | **No** |  |  |
|  | **n = 292** | **n = 3343** |  |  |
| Male n=2105 | 156 7.4% | 1969 93.5% | 0.640  (0.497-0.822) | 0.001 |
| Female n=1530 | 136 8.9% | 1374 89.8% | Ref |  |

## 6. Mortality by age and sex category by human endemic coronavirus (CoV) and high-density pneumococcus (HDSpn) NP/OP co-detection category

| **Mortality ^a^** | **No. (% with available information)** | | | |  | **Adjusted p-value^a^** | | |
| --- | --- | --- | --- | --- | --- | --- | --- | --- |
|  | **A. CoV+ / HDSpn+**  **n=43** | **B. CoV+ / HDSpn-**  **n=247** | **C. CoV- / HDSpn+**  **n=449** | **D. CoV- or HDSpn -**  **n=3149** | **Overall** | **A vs B** | **A vs C** | **A vs D** |
| **Overall** | 9 / 40 | 19 / 235 | 41 / 422 | 205 / 2864 | 0.053 | **0.006** | **0.035** | **0.006** |
|  | (22.5) | (8.1) | (9.7) | (7.2) |  |  |  |  |
| **By Sex** |  |  |  |  |  |  |  |  |
| Male | 7 / 20 | 8 / 150 | 16 / 227 | 95 / 1670 | **0.004** | **<0.001** | **0.002** | **<0.001** |
|  | (35.0) | (5.3) | (7.1) | (5.7) |  |  |  |  |
| Female | 2 / 20 | 11 / 85 | 25 / 195 | 110 / 1194 | 0.694 | 0.739 | 0.762 | 0.992 |
|  | (10.0) | (12.9) | (12.8) | (9.2) |  |  |  |  |
| **By Age Category** |  |  |  |  |  |  |  |  |
| 28 days – 11 months | 5 / 25 | 15 / 164 | 24 / 262 | 158 / 1765 | 0.304 | 0.100 | 0.089 | 0.057 |
|  | (20.0) | (9.2) | (9.2) | (9.0) |  |  |  |  |
| 12 – 59 months | 4 / 15 | 4 / 71 | 17 / 160 | 47 / 1099 | **<0.001** | **0.011** | 0.068 | **<0.001** |
|  | (26.7) | (5.6) | (10.6) | (4.3) |  |  |  |  |

a. Death within 30 days of admission. Data missing for 3/43 in CoV+/Spn+ group.

## 7. Adjusted Odds Ratios for Morbidity and Mortality for Nasopharyngeal/Oropharyngeal Co-Detection with Human Endemic Coronavirus (Cov) and High-Density *S. Pneumoniae* (HDSpn) among Children Hospitalized with Severe or Very Severe Pneumonia, Adjusted for Age, Site Region, and Malnutrition.

|  | **CoV+/HDSpn+ vs. CoV+/HDSpn-** | |  | **CoV+/HDSpn+ vs. CoV-/HDSpn+** | |  | **CoV+/HDSpn+ vs. CoV-/HDSpn-** | |
| --- | --- | --- | --- | --- | --- | --- | --- | --- |
|  | **aOR^a^**  **(95% CI)** | **p-value** |  | **aOR^a^**  **(95% CI)** | **p-value** |  | **aOR^a^**  **(95% CI)** | **p-value** |
| **Death^b^** |  |  |  |  |  |  |  |  |
| Male | 11.65  (3.05-44.43) | **<0.001** |  | 5.92  (1.72 – 20.32) | **0.005** |  | 8.61  (3.06 – 24.23) | **<0.001** |
| Female | 0.40  (0.06 - 2.88) | 0.365 |  | 0.85  (0.16 – 4.40) | 0.842 |  | 1.23  (0.27 – 5.72) | 0.788 |
| **Very severe pneumonia** |  |  |  |  |  |  |  |  |
| Male | 5.01  (1.74 – 14.47) | **0.003** |  | 2.06  (0.70 – 6.06) | 0.187 |  | 3.71  (1.46 – 9.43) | **0.006** |
| Female | 1.55  (0.50 – 4.80) | 0.445 |  | 1.51  (0.54 – 4.18) | 0.432 |  | 2.14  (0.83 – 5.51) | 0.116 |
| **Very severe pneumonia or hypoxemia** |  |  |  |  |  |  |  |  |
| Male | 6.05  (1.83 – 20.06) | **0.003** |  | 2.63  (0.77 – 8.95) | 0.123 |  | 4.14  (1.44 – 11.91) | **0.008** |
| Female | 1.54  (0.47 – 5.08) | 0.477 |  | 2.02  (0.65 – 6.23) | 0.223 |  | 2.35  (0.85 – 6.52) | 0.101 |
| **Supplemental oxygen (ever)^c^** |  |  |  |  |  |  |  |  |
| Male | 4.12  (1.30 – 13.10) | **0.016** |  | 1.55  (0.52 – 4.65) | 0.433 |  | 3.25  (1.21 – 8.69) | **0.019** |
| Female | 0.87  (0.19 – 4.13) | 0.864 |  | 1.27  (0.31 – 5.21) | 0.740 |  | 1.14  (0.31 – 4.23) | 0.845 |

CoV, Coronavirus; HDSpn, High-density *Streptococcus pneumoniae*; OR, Odds ratio; CI, Confidence interval

a. Adjusted for age, site region (Asia, West Africa, Southern Africa, and East Africa), and severe malnutrition by Z-score <-3 standard deviations.

b. Death within 30 days of admission.

c. Excludes South Africa due to near uniformity of receiving oxygen at the South Africa PERCH site.

## 8a. Case fatality ratio by NP/OP co-detection of any density *S. pneumoniae* (Spn) with human endemic coronavirus (CoV)

|  | **Co-detection Category** | | | |
| --- | --- | --- | --- | --- |
|  | **A. CoV+ / Spn+** | **B. CoV+ / Spn-** | **C. CoV- / Spn+** | **D. CoV- / Spn -** |
|  | **n=196** | **n=79** | **n=2369** | **n=917** |
| **Death** | 19 (9.7) | 9 (11.4) | 163 (6.9) | 83 (9.1) |
| Male n=2067 | 10 (8.3) | 5 (10.2) | 72 (5.3) | 39 (7.4) |
| Female n=1494 | 9 (12.0) | 4 (13.3) | 91 (9.1) | 44 (11.4) |

## 8b. Case fatality ratio by NP/OP co-detection of high (HDSpn) or low (LDSpn) density *S. pneumoniae* with coronavirus (CoV)

|  | **Mortality^a^** | | | | | |
| --- | --- | --- | --- | --- | --- | --- |
|  | **CoV+ (n=275)** | | | **Cov- (n=3287)** | | |
| ***S. pneumoniae* carriage** | **Spn-** | **LDSpn**^b^ | **HDSpn** | **Spn-** | **LDSpn** | **HDSpn** |
| **Total** | 9/79  11.4% | 10/156  6.4% | 9/40  22.5% | 83/917  9.1% | 122/1948  6.3% | 41/422  9.7% |
| **Males** | 5/49  10.2% | 3/101  3.0% | 7/20  35.0% | 39/530  7.4% | 56/1140  4.9% | 16/227  7.0% |
| **Females** | 4/30  13.3% | 7/55  12.7% | 2/20  10.0% | 44/387  11.4% | 66/808  8.2% | 25/195  12.8% |

1. Died within 30 days of hospital admission
2. Low density carriage defined as *S. pneumoniae* PCR detection <6.9 log_10_ copies/mL

## 9. Coronavirus (CoV) density (log_10_ copies/mL) by sex, mortality, and *S. pneumoniae* (Spn) co-detection status

| **CoV group** | **N** | **CoV Median Viral Load (log_10_ copies/mL)** | | | |
| --- | --- | --- | --- | --- | --- |
|  |  | **Median** | **Lower Quartile** | **Upper Quartile** | **Wilcoxon p-value** |
| **Overall CoV+** | 290 | 5.23 | 3.73 | 6.41 |  |
| Male | 180 | 5.21 | 3.76 | 6.32 | 0.127 |
| Female | 110 | 5.29 | 3.62 | 6.56 |  |
| **Co-detection with high-density Spn** | 43 | 5.78 | 3.74 | 7.15 |  |
| Male | 22 | 6.19 | 3.68 | 7.15 | 0.474 |
| Female | 21 | 5.72 | 4.22 | 6.56 |  |
| **Mortality** |  |  |  |  |  |
| Alive | 247 | 5.15 | 3.58 | 6.29 | 0.092 |
| Died^a^ | 28 | 5.74 | 4.11 | 7.37 |  |
| **Mortality among co-detection with high-density Spn** |  |  |  |  |  |
| Alive | 31 | 5.58 | 3.74 | 6.19 | **0.025** |
| Died^a^ | 9 | 7.15 | 6.27 | 7.87 |  |
| **Spn Density Category** |  |  |  |  |  |
| Non-carriers | 82 | 4.91 | 3.73 | 6.28 | 0.360 |
| Low-density carriage | 165 | 5.34 | 3.65 | 6.37 |  |
| High-density carriage (≥6.9 log_10_ copies/mL) | 43 | 5.78 | 3.74 | 7.15 |  |

a. Died within 30 days of admission

## 10. Coronavirus (CoV) viral load by high-density *S. pneumoniae* (HDSpn) co-detection status and sex


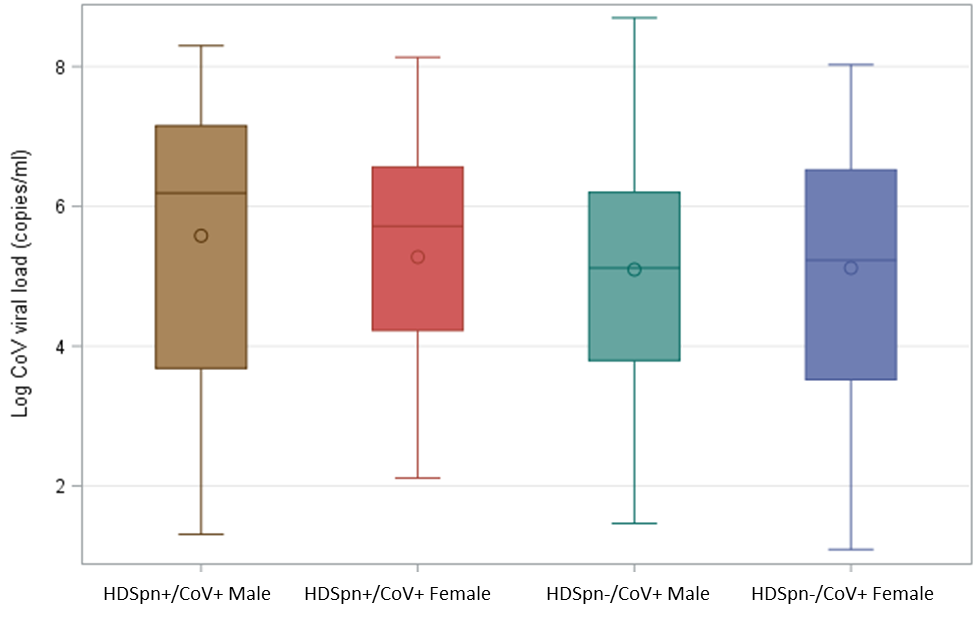


No statistically significant differences in log CoV density between groups. Median values indicated by horizontal line. Mean values indicated by circles. Quartile 1 and quartile 3 indicated by the bottom and top of box, respectively.

## 11. Clinical and etiologic findings among cases who died with human endemic coronavirus (CoV) in NP/OP

## 11a. With high-density *S. pneumoniae* (HDSpn) NP/OP co-detection

| **Site** | **Sex** | **Age (mo.)** | **Days Ill Prior to Admission (days)** | **Severity^a^** | **Clinical history and presentation^b^** | **C-reactive protein (normal: <40)** | **Chest X-ray conclusion (at admission)** | **Malnutrition^c^** | **Other comorbidities** | **Immediate and secondary cause of death from medical record** | **Pleural fluid culture** | **Blood culture** | **NP/OP PCR** | | | | | **Induced Sputum^e^ PCR** | |
| --- | --- | --- | --- | --- | --- | --- | --- | --- | --- | --- | --- | --- | --- | --- | --- | --- | --- | --- | --- |
|  |  |  |  |  |  |  |  |  |  |  |  |  | **S.pn log_10_ copies/mL** | **S.pn serotype** | **CoV log_10_ copies/mL** | **High density *H. influenzae^d^*** | **Other Organisms Detected** | ***S.pn***  **log_10_ copies/mL** | **CoV log_10_ copies/mL** |
| G | F | 6 | 2 | Very Severe | Cough, Fever, Hypoxia, Lethargy | 193.6 | Consolidation | Normal | 0 | 1. Hypoxia  2. Pneumonia | *S. aureus* | Negative | 7.6 | 6A | 7.9 | Yes | CMV,  *C. pneumoniae,*  *M. catarrhalis* | NA^f^ | NA |
| M | F | 27 | 4 | Very Severe | Cough, Diarrhea, Lethargy, Lymphopenia | Missing | Missing | WFA, WFH, HFA | 0 | 1. Shock / dehydration  2. Metabolic acidosis |  | S.pn | 8.0 | 19F | 6.6 | Yes | CMV | NA | NA |
| K | M | 12 | 14 | Very Severe | Cough, Fever, Vomiting, Diarrhea, Lethargy | 18.4 | Normal | MUAC <115, HFA | 0 | 1. Malnutrition  2. Gastroenteritis |  | Negative | 7.5 | 19F | 3.6 | Yes | Adenovirus, CMV,  Human bocavirus,  *M. catarrhalis*, Rhinovirus,  RSV | 6.29 | 0 |
| M | M | 3 | 6 | Severe | Cough, Fever, Vomiting, Diarrhea, Hypoxia, Meningitis | 3.4 | Normal | WFH, HFA | 0 | 1. Pneumonia |  | Negative | 8.5 | 6B | 8.2 | Yes | *M. catarrhalis* | NA | NA |
| M | M | 12 | 5 | Severe | Cough, Fever, Vomiting, Hypoxia | 384.4 | Consolidation with other infiltrate | WFA, WFH, HFA | 0 | 1. Shock / dehydration  2. Pneumonia |  | Negative | 7.2 | 6A | 6.3 | Yes | CMV,  *M. catarrhalis*, PV/EV | 0 | 5.43 |
| M | M | 10 | 3 | Very Severe | Cough, Fever, Vomiting, Hypoxia | 105.0 | Missing | WFA, HFA | 0 | 1. Metabolic acidosis  2. Pneumonia, malnutrition |  | Pseudomonas aeruginosa | 7.2 | NA | 7.2 | 0 | *M. catarrhalis*, rhinovirus | NA | NA |
| M | M | 6 | 7 | Very Severe | Cough, Fever, Vomiting, Diarrhea | 24.5 | Consolidation | Normal | 0 | 1. Pneumonia |  | Negative | 7.6 | 19A | 8.1 | 0 | Adenovirus, CMV,  *M. catarrhalis*, PIV 2,  Rhinovirus | NA | NA |
| M | M | 14 | 1 | Very Severe | Fever, Hypoxia | 1.9 | Missing | WFA, WFH | HIV admission diagnosis (HIV test status unknown) | 1. Pneumonia |  | Negative | 6.9 | 23F | 7.7 | 0 | CMV,  *M. catarrhalis* | NA | NA |
| Z | M | 5 | 2 | Very Severe | Cough, Fever, Lethargy, Lymphopenia | 2.7 | Uninterpretable | Normal | 0 | 1. Pneumonia  2. Meningitis, sepsis, paralytic ileus |  | Negative | 7.6 | 23B | 3.7 | 0 | CMV,  *M. catarrhalis* | NA | NA |

## 11b. Without high-density *S. pneumoniae* (HDSpn) NP/OP co-detection

| **Site** | **Sex** | **Age (mo.)** | **Days Ill Prior to Admission (days)** | **Severity^a^** | **Clinical history and presentation^b^** | **C-reactive protein (normal: <40)** | **Chest X-ray conclusion (at admission)** | **Malnutrition^c^** | **Immediate and secondary cause of death from medical record** | **Pleural fluid culture** | **Blood culture** | **NP/OP PCR** | | | | **Induced Sputum^e^ PCR** | |
| --- | --- | --- | --- | --- | --- | --- | --- | --- | --- | --- | --- | --- | --- | --- | --- | --- | --- |
|  |  |  |  |  |  |  |  |  |  |  |  | **S.pn log_10_ copies/mL** | **CoV log_10_ copies/mL** | **High density *H. influenzae^d^*** | **Other Organisms Detected** | ***S.pn***  **log_10_ copies/mL** | **CoV log_10_ copies/mL** |
| K | F | 43 | **7** | Very Severe | Fever, Lethargy | 382.6 | Consolidation |  | 1. Malaria |  | Negative | 5.4 | 7.5 |  | *M. catarrhalis* | NA | NA |
| K | F | 1 | 14 | Very Severe | Cough, Fever, Hypoxia | . | Consolidation | HFA, WFA | 1. Heart disease |  | Negative | 0 | 5.1 |  | PCP, Rhinovirus, RSV, Salmonella, *S. aureus* | 0 | 4.4 |
| G | F | 6 | 3 | Severe | Cough, Fever, Hypoxia | 20.7 | Normal | MUAC<115 | 1. Pneumonia |  | Negative | 3.6 | 6.2 |  | CMV,  *M. catarrhalis* | 0 | 4.7 |
| G | F | 16 | 8 | Very Severe | Cough, Fever, Lethargy | 87.4 | Consolidation | HFA, WFA | 1. Pneumonia |  | E.coli | 0 | 6.0 |  | *S. aureus* | 0 | 6.5 |
| G | F | 3 | 3 | Severe | Cough, Fever | 41 | Consolidation |  | 1. Pneumonia |  | Negative | 4.5 | 3.5 |  | CMV,  *M. catarrhalis,* RSV | 4.6 | 0 |
| M | F | 6 | **7** | Very Severe | Cough, Fever, Hypoxia | 100.4 | Missing |  | 1. Pneumonia |  | Negative | 5.5 | 4.4 |  | CMV,  *M. catarrhalis* | NA | NA |
| M | F | 2 | 3 | Very Severe | Cough, Fever, Vomiting | 27.3 | Missing |  | 1. Pneumonia |  | Negative | 4.3 | 2.2 |  | Human bocavirus, *M. catarrhalis,* PCP | NA | NA |
| M | F | 3 | 6 | Severe | Cough, Fever, Hypoxia, Lymphopenia, Vomiting | 3 | Uninterpretable | MUAC<115, WFA, WFH | 1. Pneumonia |  | Negative | 4.0 | 3.8 |  | CMV, PCP | 4.2 | 5.4 |
| M | F | 3 | 14 | Very Severe | Cough, Fever, Hypoxia, Vomiting, Meningitis | 0.8 | Consolidation |  | 1. Pneumonia, 2. Heart disease, Anemia |  | Negative | 0 | 3.1 |  | CMV, PCP | NA | NA |
| M | F | 8 | 3 | Severe | Cough, Fever, Vomiting, | 3.4 | Uninterpretable | MUAC<115, WFA, WFH | 1. Pneumonia, Heart disease |  | Negative | 0 | 7.2 |  | CMV, Human bocavirus,  *M. catarrhalis*, PCP, *S. aureus* | NA | NA |
| S | F | 6 | **4** | Severe | Cough, Diarrhea, Fever, Hypoxia, Vomiting | 1.8 | Consolidation |  | 1. Cardiac shock |  | Negative | 6.7 | 4.7 | Yes | *M. catarrhalis*, PCP | NA | NA |
| K | M | 0 | 1 | Severe | Hypoxia | 0.2 | Uninterpretable |  | 1. Pneumonia |  | Negative | 0 | 5.3 |  | PV/EV, *S. aureus* | NA | NA |
| M | M | 6 | 4 | Very Severe | Cough, Fever, Hypoxia, Vomiting | 47 | Normal | MUAC<115, WFA, WFH | 1. Heart disease |  | Negative | 0 | 4.7 |  |  | NA | NA |
| M | M | 28 | 3 | Very Severe | Meningitis | 23.9 | Normal | HFA, WFA, WFH | Tetanus |  | Negative | 0 | 1.5 |  | Adenovirus, CMV, *M. catarrhalis* | NA | NA |
| M | M | 12 | 4 | Very Severe | Fever, Lethargy, Meningitis | 4.9 | Normal | WFH | 1. Malaria, Meningitis |  | Negative | 4.4 | 6.0 |  | CMV, *S. aureus* | 0 | 4.2 |
| Z | M | 7 | **5** | Severe | Cough, Diarrhea, Fever, Lymphopenia | 86.2 | Normal |  | 1. Pneumonia |  | Negative | 6.8 | 8.1 | Yes | CMV,  *M. catarrhalis*, PCP | NA | NA |
| Z | M | 4 | 5 | Very Severe | Cough, Fever, Hypoxia, Lethargy | 16.8 | Uninterpretable |  | 1. Pneumonia, Septicaemia |  | Negative | 3.6 | 5.5 |  |  | NA | NA |
| S | M | 0 | 3 | Very Severe | Cough, Fever, Hypoxia, Lethargy | 70.9 | Consolidation |  | 1. Pneumonia |  | Negative | 0 | 4.5 |  | Rhinovirus, RSV | NA | NA |
| T | M | 2 | 7 | Severe | Cough, Fever, Vomiting | 2.6 | Normal |  | 1. Pneumonia |  | Negative | 0 | 7.5 | Yes | CMV, Human bocavirus,  *M. catarrhalis*, PV/EV | 0 | 7.4 |

1. Severe vs. very severe pneumonia defined using pre-2013 WHO classification. Severe pneumonia was defined as having cough or difficulty breathing and lower chest wall indrawing; very severe pneumonia was defined as cough or difficulty breathing and at least one of the following: central cyanosis, difficulty breastfeeding/drinking, vomiting everything, convulsions, lethargy, unconsciousness or head nodding.
2. Including cough, fever, vomiting, diarrhea, lethargy, hypoxia, lymphopenia, and meningitis.
3. Malnutrition listed if patients are severe (<-3 Z score).
4. High density *H. influenzae* > 5.9 log10 copies/mL.
5. Mycobacterium tuberculosis was tested for using induced sputum culture and was not detected among the listed patients.
6. Induced sputum not collected because patient died or was deemed to be too severe to undergo sampling procedure.

Abbreviations: site: G=Gambia, K=Kenya, M=Mali, S=South Africa, T=Thailand, Z=Zambia; sex: F=female, M=male; NA = not available; organisms: CoV=coronavirus, CMV=cytomegalovirus, PCP=pneumocystis jirovecii, PIV=parainfluenza virus, PV/EV= parechovirus/enterovirus, RSV=respiratory syncytial virus, S.pn = S. pneumoniae; malnutrition: MUAC=middle upper arm circumference, HFA=height for age, WFA=weight for age, WFH=weight for height.

## 12. High-density pneumococcus (HDSpn) detection and pneumococcal load in clinical samples by co-detection category with human endemic coronavirus (CoV)

| **Cases** | **No. with available information (col %)** | | | |
| --- | --- | --- | --- | --- |
|  | **Co-detection Category** | | | |
|  | **A. CoV+ / HDSpn+** | **B. CoV+ / HDSpn-** | **C. CoV- / HDSpn+** | **D. CoV- / HDSpn -** |
|  | **n=43** | **n=247** | **n=449** | **n=3149** |
| **Invasive pneumococcal disease^a^** | 4 | 3 | 23 | 14 |
|  | (9.5) | (1.2) | (5.2) | (0.5) |
| Lung aspirate PCR | 1 / 1 | 0 / 0 | 3 / 8 | 1 / 17 |
|  | (100.0) | . | (37.5) | (5.9) |
| Lung aspirate culture | 0 / 2 | 0 / 2 | 3 / 11 | 1 / 27 |
|  | - | - | (27.3) | (3.7) |
| Pleural fluid PCR | 0/0 | 1 / 1 | 0 / 2 | 4 / 13 |
|  | - | (100) | - | (30.8) |
| Pleural fluid culture | 0 / 1 | 1 / 1 | 0 / 4 | 0 / 15 |
|  | - | (100) | - | - |
| Blood culture | 3 | 3 | 19 | 8 |
|  | (7.1) | (1.2) | (4.3) | (0.3) |
| **Blood lytA PCR** | 3 | 15 | 56 | 174 |
|  | (7.7) | (6.4) | (13.0) | (5.9) |
| **Blood lytA PCR > 2.2 log copies/mL** | 3 | 13 | 45 | 111 |
|  | (7.7) | (5.5) | (10.4) | (3.7) |
| **Induced sputum culture** | 14 | 63 | 192 | 772 |
|  | (41.2) | (28.8) | (49.2) | (27.0) |
| **Induced sputum PCR** | 28 | 132 | 368 | 1849 |
|  | (93.3) | (62.9) | (96.1) | (65.3) |
| **Nasopharyngeal culture** | 37 | 117 | 400 | 1510 |
|  | (88.1) | (48.0) | (90.5) | (48.4) |
| ***S. pneumoniae* (median log10 copies/mL)** | 7.24 | 4.50 | 7.23 | 4.67 |
| IQR | 7.14-7.54 | 0-5.96 | 7.08-7.52 | 0-5.95 |

a. *Streptococcus pneumoniae* detected on blood culture, lung aspirate PCR, lung aspirate culture, pleural fluid culture, or pleural fluid PCR

## 13. Median number of pathogens positive on NP/OP PCR by human endemic coronavirus (CoV) and high-density pneumococcus (HDSpn) co-detection category, among cases

| **Cases** | **Co-detection Category** | | | |  |
| --- | --- | --- | --- | --- | --- |
|  | **A. CoV+ / HDSpn+** | **B. CoV+ / HDSpn-** | **C. CoV- / HDSpn+** | **D. CoV- and HDSpn -** | **p-value** |
|  | **n=43** | **n=247** | **n=449** | **n=3149** |  |
| **Number of pathogens** | 5.0 | 4.0 | 4.0 | 2.0 | <0.001 |
| Male | 5.0 | 4.0 | 4.0 | 2.0 |  |
| Female | 5.0 | 3.0 | 4.0 | 2.0 |  |

## 14. Sensitivity analyses evaluating association of clinical outcomes by co-detection group including additional CoV and Spn cases

## 14a. Adding microbiologically confirmed pneumococcal pneumonia^a^ to high density *S. pneumoniae* (HDSpn) and adding human endemic CoV detected in induced sputum to cases with CoV detected in NP/OP

|  | **Co-detection Category** | | | | **Adjusted Odds Ratio^b^** | | | **p-value^b^** | | | |
| --- | --- | --- | --- | --- | --- | --- | --- | --- | --- | --- | --- |
|  | **A. CoV+ / HDSpn+** | **B. CoV+ / HDSpn-** | **C. CoV- / HDSpn+** | **D. CoV- and HDSpn -** | **A v B** | **A v C** | **A v D** | **Overall** | **AvB** | **AvC** | **AvD** |
|  | **n=53** | **n=337** | **n=388** | **n=2730** |  |  |  |  |  |  |  |
| **Very severe pneumonia (WHO Definition)** | 25  47.2 | 106  31.5 | 133  34.3 | 765  28.0 |  |  |  |  |  |  |  |
| Male | 14  51.9 | 66  30.0 | 67  31.5 | 438  27.4 | 2.8  1.1-7.0 | 2.3  0.9-6.0 | 2.8  1.2-6.7 | **0.038** | **0.032** | 0.092 | **0.017** |
| Female | 11  42.3 | 40  34.2 | 66  37.7 | 327  28.8 | 1.4  0.5-3.7 | 1.5  0.6-3.8 | 1.9  0.8-4.3 | **0.032** | 0.526 | 0.394 | 0.148 |
| **WHO Defined Very Severe or Hypoxic** | 33  62.3 | 170  50.6 | 208  53.6 | 1308  48.0 |  |  |  |  |  |  |  |
| Male | 17  63.0 | 106  48.2 | 104  48.8 | 731  45.9 | 2.2  0.8-6.0 | 2.1  0.7-5.8 | 2.5  1.0-6.4 | **0.010** | 0.118 | 0.178 | 0.055 |
| Female | 16  61.5 | 64  55.2 | 104  59.4 | 577  50.9 | 1.6  0.6-4.4 | 1.8  0.6-5.0 | 2.2  0.9-5.5 | **0.039** | 0.379 | 0.274 | 0.100 |
| **Supplemental oxygen (ever)^b^** | 15  32.6 | 66  24.8 | 76  24.2 | 517  24.3 |  |  |  |  |  |  |  |
| Male | 10  40.0 | 37  20.9 | 45  25.0 | 289  22.9 | 4.1  1.4-12.0 | 1.9  0.7-5.3 | 3.4  1.3-8.6 | **0.001** | **0.009** | 0.240 | **0.010** |
| Female | 5  23.8 | 29  32.6 | 31  23.1 | 228  26.4 | 0.9  0.3-2.8 | 2.1  0.7-7.0 | 2.6  0.8-7.7 | 0.153 | 0.809 | 0.210 | 0.095 |
| **Observed Cough** | 25  47.2 | 236  70.5 | 276  71.3 | 1976  72.6 |  |  |  |  |  |  |  |
| Male | 11  40.7 | 157  71.7 | 154  72.3 | 1164  73.1 | 3.0  1.2-7.5 | 0.3  0.1-0.7 | 3.6  1.6-8.3 | **0.038** | **0.017** | **0.007** | **0.003** |
| Female | 14  53.9 | 79  68.1 | 122  70.1 | 812  71.9 | 1.6  0.6-4.5 | 2.0  0.8-5.0 | 2.0  0.9-4.6 | 0.328 | 0.346 | 0.144 | 0.111 |
| **Abnormal Chest X-ray*** | 20  52.6 | 144  49.2 | 205  60.7 | 1192  50.1 |  |  |  |  |  |  |  |
| Male | 14  70.0 | 84  43.3 | 109  59.6 | 690  49.2 | 3.6  1.2-9.9 | 1.4  0.5-4.1 | 2.3  0.9-6.0 | **0.002** | **0.015** | 0.483 | 0.094 |
| Female | 6  33.3 | 60  60.6 | 96  61.9 | 502  51.4 | 0.3  0.1-1.0 | 0.3  0.1-0.8 | 0.5  0.2-1.4 | **0.002** | 0.061 | **0.021** | 0.186 |
| **Severe Acute Malnutrition^d^** | 9  17.0 | 56  16.9 | 74  19.3 | 436  16.1 |  |  |  |  |  |  |  |
| Male | 7  25.9 | 34  15.5 | 41  19.4 | 231  14.6 | 1.8  0.7-5.0 | 1.4  0.5-3.7 | 1.8  0.7-4.4 | 0.348 | 0.257 | 0.514 | 0.186 |
| Female | 2  7.7 | 22  19.5 | 33  19.1 | 205  18.3 | 0.4  0.1-1.8 | 0.3  0.1-1.5 | 0.3  0.1-1.5 | 0.557 | 0.229 | 0.143 | 0.159 |
| **Height-for-Age Z-Score < -3 SDs** | 10  21.3 | 46  16.3 | 52  16.6 | 430  18.7 |  |  |  |  |  |  |  |
| Male | 7  28.0 | 34  18.1 | 25  14.8 | 273  20.3 | 2.1  0.7-6.0 | 2.7  0.8-8.6 | 2.3  0.9-5.9 | 0.327 | 0.171 | 0.095 | 0.098 |
| Female | 3  13.6 | 12  12.8 | 27  18.6 | 157  16.4 | 1.6  0.3-7.8 | 1.1  0.3-4.6 | 1.4  0.4-5.1 | 0.162 | 0.585 | 0.898 | 0.575 |
| **C-Reactive Protein (mg/L) (median, IQR)** | 43.7  (8.0 – 185.9) | 13.5  (3.9 – 41.0) | 26.7  (7.0 – 96.7) | 12.6  (3.4 – 37.8) |  |  |  |  |  |  |  |
| Male | 43.7  (7.8 – 258.5) | 13.2  (4.6 – 38.6) | 22.0  (5.9 – 84.9) | 12.6  (3.0 – 37.6) |  |  |  | **<0.001** | **<0.001** | 0.053 | **<0.001** |
| Female | 43.5  (8.1 – 178.2) | 15.7  (3.3 – 48.9) | 30.4  (11.1 – 110.1) | 12.8  (3.8 – 38.1) |  |  |  | **<0.001** | 0.261 | 0.809 | **0.031** |
| **Underlying Condition^e^** | 5  9.4 | 41  12.2 | 40  10.3 | 312  11.4 |  |  |  |  |  |  |  |
| Male | 4  14.8 | 24  10.9 | 15  7.0 | 168  10.5 | 1.9  0.6-6.5 | 2.0  0.6-7.3 | 2.0  0.7-6.1 | 0.457 | 0.305 | 0.286 | 0.206 |
| Female | 1  3.9 | 17  14.5 | 25  14.3 | 144  12.7 | 0.02  0.0-2.0 | 0.3  0.0-2.4 | 0.4  0.1-3.3 | 0.353 | 0.179 | 0.246 | 0.420 |
| **Death^f^** | 9/50  18.0 | 23/319  7.2 | 15/364  4.1 | 71/2478  2.9 |  |  |  |  |  |  |  |
| Male | 7/25  28.0 | 11/207  5.3 | 5/199  2.5 | 31/1459  2.1 | 8.4  2.3-30.7 | 15.3  4.4-53.6 | 15.8  5.6-44.9 | **<0.001** | **0.001** | **<0.001** | **<0.001** |
| Female | 2/25  8.0 | 12/112  10.7 | 10/165  6.1 | 40/1019  3.9 | 0.5  0.1-2.7 | 1.4  0.3-7.1 | 2.6  0.6-11.9 | **0.048** | 0.443 | 0.649 | 0.226 |

## 14b. Lowering the threshold to >6.6 log10 copies/mL to define high density *S. pneumoniae* (HDSpn)

|  | **Co-detection Category** | | | | **Adjusted Odds Ratio^b^** | | | **p-value^b^** | | | |
| --- | --- | --- | --- | --- | --- | --- | --- | --- | --- | --- | --- |
|  | **A. CoV+ / HDSpn+** | **B. CoV+ / HDSpn-** | **C. CoV- / HDSpn+** | **D. CoV- and HDSpn -** | **A v B** | **A v C** | **A v D** | **Overall** | **AvB** | **AvC** | **AvD** |
|  | **n=64** | **n=226** | **n=697** | **n=2902** |  |  |  |  |  |  |  |
| **Death^f^** |  |  |  |  |  |  |  |  |  |  |  |
| Male | 8/34  23.5 | 7/136  5.2 | 22/351  6.3 | 89/1546  5.8 | 5.7  1.5-21.4 | 4.7  1.9-11.6 | 3.8  1.5-9.3 | **<0.001** | **0.009** | **0.001** | **0.004** |
| Female | 3/26  11.5 | 10/79  12.7 | 33/296  11.2 | 102/1094  9.3 | 0.6  0.1-2.5 | 1.1  0.3-4.2 | 1.1  0.3-4.2 | **0.033** | 0.465 | 0.870 | 0.842 |

a. Pneumococcus detected from a normally sterile site

b. P-values for pairwise comparisons of categorical variables obtained from logistic regression model adjusted for age and site (only age adjusted for A vs C comparison on death due to sample size limitations). Overall p-value obtained from multinomial logistic regression adjusted for age and site (where applicable).

c. Excludes South Africa due to near uniformity of receiving oxygen at the South Africa PERCH site.

d. Severe acute malnutrition

e. Underlying conditions: cerebral palsy, congenital heart disease/defect, congenital abnormalities, developmental delay, severe malnutrition, prematurity in an infant <6 months old. f. The number of days with cough, fever, difficulty breathing, wheeze, or runny nose, whichever symptom is longest.

f. Death within 30 days of admission

## 15. Case fatality risk by NP/OP co-detection of other pathogen combinations, by sex

## 15a. High-density *H. influenzae* (HDHinf) and any coronavirus (CoV)

|  | **Co-detection Category** | | | |  |
| --- | --- | --- | --- | --- | --- |
|  | **CoV+ / HDHinf+** | **CoV+ / HDHinf-** | **CoV- / HDHinf+** | **CoV- and HDHinf -** | **p-value** |
|  | **n=84** | **n=191** | **n=896** | **n=2390** |  |
| **Death** | 8 (9.5) | 20 (10.5) | 60 (6.7) | 186 (7.8) | 0.290 |
| Male n=2067 | 5/55 (9.1) | 10/115 (8.7) | 27/512 (5.3) | 84/1385 (6.1) | 0.419 |
| Female n=1494 | 3/29 (10.3) | 10/76 (13.2) | 33/384 (8.6) | 102/1005 (10.2) | 0.634 |

## 15b. *S. aureus* (Saur) and any coronavirus (CoV)

|  | **Co-detection Category** | | | |  |
| --- | --- | --- | --- | --- | --- |
|  | **CoV+ / Saur+** | **CoV+ / Saur-** | **CoV- / Saur+** | **CoV- and Saur -** | **p-value** |
|  | **n=45** | **n=230** | **n=523** | **n=2763** |  |
| **Death** | 5  (11.1) | 23  (10.0) | 71  (13.6) | 175  (6.3) | **<0.001** |
| Male n=2067 | 2/29  (6.9) | 13/141  (9.2) | 36/299  (12.0) | 75/1598  (4.7) | **<0.001** |
| Female n=1494 | 3/16  (18.8) | 10/89  (11.2) | 35/224  (15.6) | 100/1165  (8.6) | **0.007** |

## 15c. High-density *S. pneumoniae* (HDSpn) and any influenza A, B or C (Flu)

|  | **Co-detection Category** | | | |  |
| --- | --- | --- | --- | --- | --- |
|  | **Flu+ / HDSpn+** | **Flu+ / HDSpn-** | **Flu- / HDSpn+** | **Flu- and HDSpn -** | **p-value** |
|  | **n=22** | **n=144** | **n=440** | **n=2972** |  |
| **Death** | 0 - | 11 7.6 | 50 11.4 | 214 7.2 | **0.011** |
| Male n=2075 | 0/10 - | 7/85 8.2 | 23/237 9.7 | 96/1743 5.5 | 0.050 |
| Female n=1503 | 0/12 - | 4/59 6.8 | 27/203 13.3 | 118/1229 9.6 | 0.194 |

## 15d. High-density *S. pneumoniae* (HDSpn) and HMPV

|  | **Co-detection Category** | | | |  |
| --- | --- | --- | --- | --- | --- |
|  | **HMPV+ / HDSpn+** | **HMPV+ / HDSpn-** | **HMPV- / HDSpn+** | **HMPV- and HDSpn -** | **p-value** |
|  | **n=35** | **n=203** | **n=427** | **n=2895** |  |
| **Death** | 2 5.7 | 8 3.9 | 48 11.2 | 216 7.5 | **0.007** |
| Male n=2066 | 1/17 5.9 | 3/117 2.6 | 22/230 9.6 | 100/1702 5.9 | 0.057 |
| Female n=1494 | 1/18 5.6 | 5/86 5.8 | 26/197 13.2 | 116/1193 9.7 | 0.218 |

## 15e. High-density *S.pneumoniae* (HDSpn) and RSV A/B

|  | **Co-detection Category** | | | |  |
| --- | --- | --- | --- | --- | --- |
|  | **RSV+ / HDSpn+** | **RSV+ / HDSpn-** | **RSV- / HDSpn+** | **RSV- and HDSpn -** | **p-value** |
|  | **n=130** | **n=810** | **n=332** | **n=2289** |  |
| **Death** | 4 3.1 | 20 2.5 | 46 13.9 | 104 8.9 | **<0.001** |
| Male n=2067 | 2/69 2.9 | 9/488 1.8 | 21/178 11.8 | 94/1332 7.1 | **<0.001** |
| Female n=1494 | 2/61 3.3 | 11/322 3.4 | 25/154 16.2 | 110/957 11.5 | **<0.001** |

## 15f. High-density *S.pneumoniae* (HDSpn) and Parainfluenza 1 or 3 (Para1/3)

|  | **Co-detection Category** | | | |  |
| --- | --- | --- | --- | --- | --- |
|  | **Para1/3+ / HDSpn+** | **Para1/3+ / HDSpn-** | **Para1/3- / HDSpn+** | **Para1/3- and HDSpn -** | **p-value** |
|  | **n=41** | **n=258** | **n=421** | **n=2841** |  |
| **Death** | 7 | 12 | 43 | 212 | **0.006** |
|  | 17.1 | 4.7 | 10.2 | 7.5 |  |
| Male n=2067 | 2/25 | 4/152 | 21/222 | 99/1668 | 0.050 |
|  | 8.0 | 2.6 | 9.5 | 5.9 |  |
| Female n=1494 | 5/16 | 8/106 | 22/199 | 113/1173 | **0.027** |
|  | 31.3 | 7.6 | 11.1 | 9.6 |  |

## 16. Mortality by coronavirus species and gender by human endemic coronavirus (CoV) and high-density pneumococcus (HDSpn) NP/OP co-detection category

| **CoV Species** |  | **Case Fatality Risk by Co-detection Category** | | **p-value** |
| --- | --- | --- | --- | --- |
|  |  | **CoV+ / HDSpn+** | **CoV- and HDSpn -** |  |
| CoV OC43 | **Overall** | 5 / 19 (26.3) | 217 / 3012 (7.2) | **0.001** |
|  | Male | 3 / 9 (33.3) | 100 (5.7) | **<0.001** |
|  | Female | 2 / 10 (20.0) | 117 (9.4) | 0.254 |
| CoV HKU1 | **Overall** | 2 / 8 (25.0) | 219 / 3012 (7.2) | 0.052 |
|  | Male | 2 / 6 (33.3) | 101 (5.7) | **0.004** |
|  | Female | 0 / 2 - | 118 (9.4) | 0.650 |
| CoV 229E | **Overall** | 0 - | 220 / 3060 (7.2) | 0.578 |
|  | Male | 0 / 1 - | 102 (5.7) | 0.806 |
|  | Female | 0 / 3 - | 118 (9.3) | 0.579 |
| CoV NL63 | **Overall** | 2 / 9 (22.2) | 220 / 3031 (7.3) | 0.085 |
|  | Male | 2 / 4 (50.0) | 101 (5.7) | **<0.001** |
|  | Female | 0 / 5 - | 119 (9.5) | 0.469 |
